# Modeling the Effects of Severe Metabolic Disease by Genome Editing of hPSC-Derived Endothelial Cells Reveals an Inflammatory Phenotype

**DOI:** 10.3390/ijms20246201

**Published:** 2019-12-09

**Authors:** Filip Roudnicky, Yanjun Lan, Max Friesen, Gregor Dernick, Jitao David Zhang, Andreas Staempfli, Natalie Bordag, Antje Wagner-Golbs, Klaus Christensen, Martin Ebeling, Martin Graf, Mark Burcin, Claas Aiko Meyer, Chad A Cowan, Christoph Patsch

**Affiliations:** 1 Roche pRED (Pharmaceutical Research and Early Development), Roche Innovation Center Basel, F.Hoffmann-La Roche Ltd., CH-4070 Basel, Switzerland; filip.roudnicky@roche.com (F.R.); yanjun.lan@riken.jp (Y.L.); gregor.dernick@roche.com (G.D.); jitao_david.zhang@roche.com (J.D.Z.); andreas.staempfli@roche.com (A.S.); Klaus.Christensen@roche.com (K.C.); martin.ebeling@roche.com (M.E.); martin.graf@roche.com (M.G.); mark.burcin@roche.com (M.B.); claas_aiko.meyer@roche.com (C.A.M.); 2 Department of Medicine, Division of Cardiology, Beth Israel Deaconess Medical Center (BIDMC), Harvard Medical School, Boston, MA 02215, USA; maxfriesen@gmail.com; 3 Metanomics Health—A BASF Group Company, 10589 Berlin, Germany; natalie@bordag.com (N.B.); a.wagner-golbs@codon.de (A.W.-G.); 4 Department of Stem Cell and Regenerative Biology, Harvard University, Cambridge, MA 02138, USA; 5 Harvard Stem Cell Institute, Harvard University, Cambridge, MA 02138, USA

**Keywords:** AKT2, genome editing, pluripotent-stem-cell-derived endothelial cells, inflammation, endothelial dysfunction

## Abstract

The kinase AKT2 (PKB) is an important mediator of insulin signaling, for which loss-of-function knockout (KO) mutants lead to early onset diabetes mellitus, and dominant active mutations lead to early development of obesity and endothelial cell (EC) dysfunction. To model EC dysfunction, we used edited human pluripotent stem cells (hPSCs) that carried either a homozygous deletion of AKT2 (AKT2 KO) or a dominant active mutation (AKT2 E17K), which, along with the parental wild type (WT), were differentiated into ECs. Profiling of EC lines indicated an increase in proinflammatory and a reduction in anti-inflammatory fatty acids, an increase in inflammatory chemokines in cell supernatants, increased expression of proinflammatory genes, and increased binding to the EC monolayer in a functional leukocyte adhesion assay for both AKT2 KO and AKT2 E17K. Collectively, these findings suggest that vascular endothelial inflammation that results from dysregulated insulin signaling (homeostasis) may contribute to coronary artery disease, and that either downregulation or upregulation of the insulin pathway may lead to inflammation of endothelial cells. This suggests that the standard of care for patients must be expanded from control of metabolic parameters to include control of inflammation, such that endothelial dysfunction and cardiovascular disorders can ultimately be prevented.

## 1. Introduction

In human tissues, insulin sensitivity is regulated through the activation of the serine/threonine kinase AKT2 (PKB beta). A loss-of-function mutation in the *AKT2* gene is the cause of a subtype of the rare disease familial partial lipodystrophy that results in severe insulin resistance and leads to early onset diabetes mellitus with lipodystrophy and hyperinsulinemia [1]. Patients that carry a partial loss-of-function variant of AKT2 tend to have a higher level of fasting plasma insulin and an increased risk of developing diabetes mellitus [2]. A gain-of-function missense mutation in AKT2, Glu17Lys (E17K), is the cause of a hypoinsulinemic hypoketotic hypoglycemia, which is also a rare genetic disease in which there is constitutive expression of AKT2, leading to severe hypoglycemia, hypoinsulinemia, and increased body fat [3]. Previously, we used genome editing in the embryonic stem cell (ESC) line HUES9 to generate an allelic series of isogenic cell lines carrying wild-type (WT) AKT2, a homozygous knockout (KO) of AKT2, or a heterozygous AKT2 E17K mutation [4].

In the present work, we focused on the effects of these AKT2 mutations on endothelial cells (ECs). ECs play a central role in the cardiovascular, renal, or neural complications of diabetes mellitus and metabolic syndrome [5]. ECs are an important target of insulin [6,7], and the primary effect of insulin is to activate the kinase AKT1, which then leads to phosphorylation of eNOS and vasodilatation to increase nutrient delivery to tissues [8]. The specific function of the closely related kinase AKT2 in endothelial cells has not been studied.

## 2. Results

**Figure 1:**
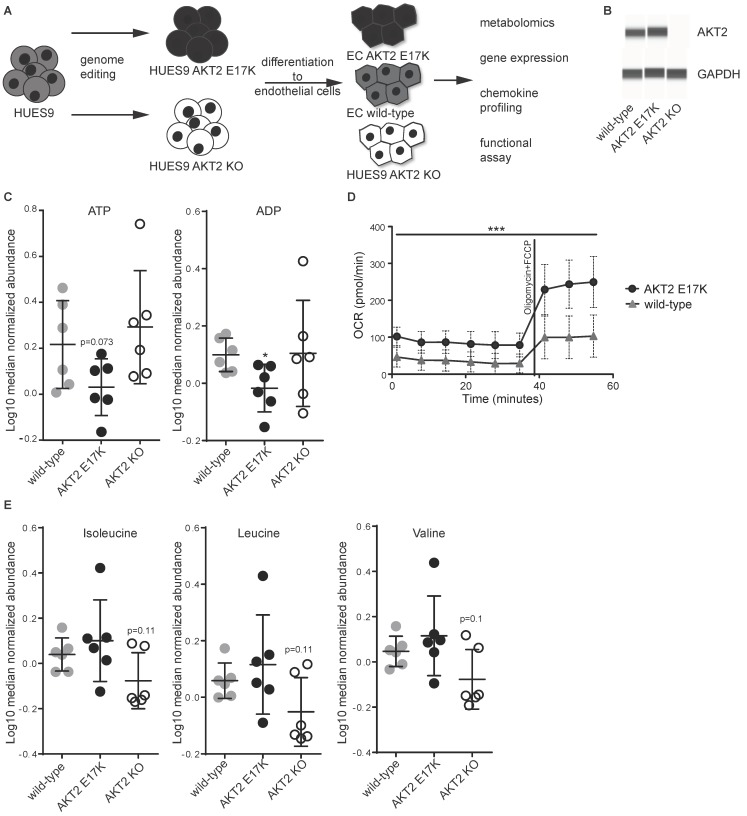
Metabolic dysregulation of human pluripotent stem cell (hPSC) endothelial cells (ECs) carrying AKT2 mutations. (**A**) Schematic representation of the engineered endothelial cells and a list of the subsequent experimentation. (**B**) Western blot of AKT2 and GAPDH from hPSC-EC cell lysates. (**C**) Abundance of ATP and ADP from six replicates of each hPSC-EC as measured by mass spectrometry from cellular lysates ± SD. (**D**) Cellular oxygen consumption rate under basal conditions and after stimulation with oligomycin (2 μM) and carbonyl cyanide-p-trifluoromethoxyphenylhydrazone (FCCP) (0.5 μM) of AKT2 wild type (WT) and AKT2 E17K hPSC-ECs. Data are presented as mean ± SD of 10 measurements per group per timepoint. (**E**) Abundance of branched-chain amino acids (BCAAs) from six replicates of each hPSC-EC as measured by mass spectrometry ± SD. For all experiments in this figure, * *p* < 0.05, *** *p* < 0.001.

To explore the effects of AKT2 dysregulation on endothelial cells, we utilized previously engineered human pluripotent stem cell (hPSC) HUES9 cell lines carrying AKT2 KO and AKT2 E17K mutations [4], along with the corresponding WT cell line, and differentiated each into ECs using a previously published protocol [7,9]. These ECs were then subjected to both molecular profiling studies and functional assays (Figure 1A). The number and percentage of ECs that were generated from stem cells did not differ between genotypes and were comparable to previously published differentiations [9]. Furthermore, the expression of PECAM1, NOTCH1, and NOS3 (Supplementary Figure S1A) were comparable between different genotypes, suggesting that the mutations did not affect the differentiation process. Western blot analysis using capillary electrophoresis confirmed that the AKT2 protein was expressed in both WT and the E17K mutant but was absent in AKT2 KO ECs (Figure 1B). Importantly, AKT1 mRNA expression did not change as a consequence of KO of AKT2 or the AKT2 E17K (Supplementary Figure S1B). Next, metabolic profiling was carried out to measure 170 metabolites in cell lysates (Supplementary Table S1) and 102 metabolites in the media supernatant (Supplementary Table S2). We identified a marked number of dysregulated metabolites, particularly in cell lysates (Table 1). A comparison of metabolic rates suggested a tendency for increased catabolism of ATP and ADP (Figure 1C) and of glucose-6-P and glycerol in AKT2 E17K cells compared with WT (Figure S2A). To validate the increase in energy demand, we performed a mitochondrial respiration assay that confirmed that AKT2 E17K cells have a higher energy demand than WT cells and showed an even greater difference when cells were challenged with the respiration inhibitors oligomycin and carbonyl cyanide-p-trifluoromethoxyphenylhydrazone (FCCP) (Figure 1D). Our results with AKT2 KO cells showed elevated levels of glucose-6-phosphate, glycerol, and glycerol-3-P (Supplementary Figure S2A). ECs of both genotypes showed a significant increase of expression of glucose transporter GLUT4 (Figure S1C).

**Figure 2:**
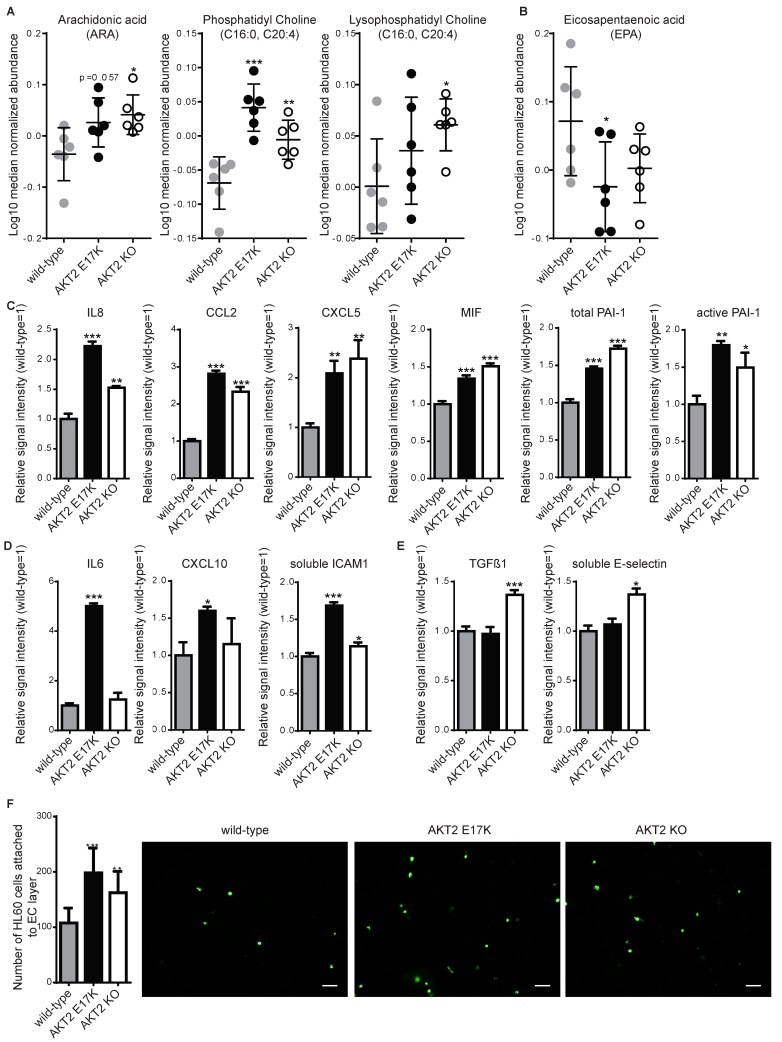
Inflammation is induced in hPSC-ECs carrying either of the two AKT2 mutations. For each experiment, hPSC-ECs with AKT2 mutations (AKT2 E17K and AKT2 knockout (KO)) were compared to WT. (**A**) Abundance of proinflammatory lipids from six replicates of each hPSC-EC and (**B**) an anti-inflammatory lipid, eicosapentaenoic acid, as measured by mass spectrometry in cellular lysates ± SD. (**C**–**E**) Inflammatory mediators measured using a multiplexed sandwich immunoassay and compared to WT with (**C**) showing those induced in both AKT2 E17K and AKT2 KO, (**D**) those induced only in AKT2 E17K, and (**E**) those induced only in AKT2 KO. (**F**) Quantification of leukocyte-like cells (HL-60) adhering to hPSC-ECs presented as the mean of triplicate experiments ± SD. * *p* < 0.05, ** *p* < 0.01, *** *p* < 0.001. Representative images for each cell line are shown.

From the total list of metabolites analyzed, the amino-acid-related group and the complex lipids and fatty acids group were most significantly affected (Table 2). Interestingly, we observed a tendency of increased catabolism of several amino acids, including the branched-chain amino acids (BCAAs) isoleucine, leucine, and valine (Figure 1E), as well as arginine, tryptophan, glycine, proline, and lysine (with varying degrees of significance; Supplementary Figure S2B) in the AKT2 KO ECs. Metabolic profiling of fatty acids showed that both of the AKT2 mutants had higher levels of proinflammatory fatty acids, such as the omega-6 polyunsaturated fatty acids (Figure 2A), and lower levels of an anti-inflammatory omega-3 polyunsaturated fatty acid (Figure 2B). The proinflammatory phenotype was confirmed using a multiplexed sandwich immunoassay and measuring elevated levels of chemokines in the supernatant from both mutant cell types (Figure 2C). Using qRT-PCR, we measured a significant increase in expression of S100A4 (a known inducer of NF-kB [10]) and CCL5 mRNA, two important inflammatory genes in ECs (Supplementary Figure S3A). AKT2 E17K had increased levels of IL-6, CXCL10, and soluble ICAM1 and decreased expression of the anti-inflammatory adrenomedullin mRNA (Supplementary Figure S3B). In AKT2 KO ECs, we measured elevated levels of TGF-ß1 and soluble SELE (Figure 2E), and qRT-PCR analysis indicated increased expression of PTGS2 mRNA (Supplementary Figure S3C).

The results of a static leukocyte adhesion assay showed a higher level of adhesion to a confluent layer of either of the mutants than to WT (Figure 2F), although to a lower extent than TNF-α-mediated inflammation (4–6-fold, Supplementary Figure S4A). To test other relevant in vitro endothelial cell functions, we evaluated the influence of AKT2 mutation/deletion on cell proliferation and migration and observed little to no difference when cells were tested under two different conditions: starvation or starvation followed by exposure to a growth factor (vascular endothelial growth factor A, VEGFA). Assays included an electrical cell impedance sensing (ECIS) assay (Supplementary Figure S4B), a 4-methylumbelliferyl heptanoate (MUH) assay for cell viability (Supplementary Figure S4C), and a test for cell migration using a monolayer migration TScratch assay [11] (Supplementary Figure S4D) or tube capillary formation (Supplementary Figure S4E). There was no significant difference between WT and the two mutant ECs in any of these four assays.

## 3. Discussion

Pluripotent stem cell technology offers the possibility of generating every cell type in the human body and, along with genome editing, has the potential to model each and every genetic disease. Although attempts have been made to model the effects of metabolic disease [12], including diabetes on mesenchymal progenitors [13] and cardiomyocytes [14] using hPSCs from diabetic patients, although previously discussed [15], no studies have explored the effects of diabetes or metabolic syndrome on endothelial cells. This is surprising, as it is known that the vascular dysfunction caused by diabetes is a prerequisite for the development of cardiovascular disease [16] and could be a critical target for the prevention of atherosclerosis. Our study aimed to understand endothelial dysfunction by taking advantage of our previously established protocol [9,17] for generating ECs from PSCs and genome editing to create models of rare metabolic diseases with AKT2 dysregulation in vitro. Patients with inactivating mutations of AKT2 develop hyperinsulinemia, hypodystrophy, and early onset diabetes [1], and we used metabolic profiling of AKT2 KO hPSC-ECs to show the expected decrease in glucose catabolism (higher levels of G6P and a tendency toward higher ATP levels). It is plausible that in the absence of AKT2, ECs compensate for the lack of glucose catabolism by catabolizing amino acids, which is in line with our observed tendency for an increased catabolism of several amino acids. Catabolism of BCAAs increases cellular levels of glutamate [18], which we observed (Supplementary Table S1), but also increases levels of 3-hydroxy-isobutyrate [19], an important inducer of lipid import into endothelial cells [20] and an inducer of inflammation. On the other hand, patients carrying the activating mutation AKT2 E17K develop hypoglycemia, hypoinsulinemia, and increased body fat, which are clinical parameters similar to those in patients who have type I diabetes. Metabolic profiling of our EC model of this mutation demonstrated similarly increased catabolism (reduced levels of ATP, ADP, G6P, and glycerol [3]).

Most strikingly, our work has revealed a cell-autonomous inflammatory phenotype when AKT2 levels are perturbed. In both of the mutant ECs, there were higher levels of arachidonic acids (unsaturated omega-6 fatty acid), which are known to increase the production of proinflammatory prostaglandins [21]. Furthermore, AKT2 KO ECs showed increased expression of PTGS2 (COX2), an enzyme that converts arachidonic acid to PGE2 [22], which are proinflammatory prostaglandins [21]. Additionally, in both mutant ECs, there was a decrease in eicosapentaenoic acid (unsaturated omega-3 fatty acid), an anti-inflammatory fatty acid that is known to reduce binding of monocytes to ECs and reduce sICAM1 expression [23]. Moreover, we measured increased levels of chemokines known to be present in atherosclerotic lesions [24] and increased adhesion of leukocytes, but this did not influence cellular proliferation, tube formation or migration behavior. Importantly, our data suggest that these engineered cell lines recapitulate several clinical parameters in vitro. Induction of PAI-1 (total and active), IL-8, CCL2, and E-selectin in AKT2 KO cells is consistent with hyperinsulinemia and hyperglycemia in patients or with experimental animal models with diabetes [25] and promotes recruitment of immune cells that stimulate inflammation. Interestingly, patients with the AKT2 E17K mutation have hypoglycemia, which is also a symptom of type I diabetes [26]. Interestingly, our EC model showed an increase in CXCL10, ICAM1, IL-6, and MIF, which are associated with type I diabetes development [27].

While further studies are necessary to understand the mechanism behind the inflammation that occurs as a result of AKT2 pathway dysregulation, our findings may be successfully extrapolated to the context of clinical diabetes as we provide evidence that both hyperinsulinemia and hypoinsulinemia cause an inflammatory phenotype that may be responsible for endothelial dysfunction. Here, we report that both knockout (AKT2 KO) and gain-of-function (AKT2 E17K) mutations lead to inflammation in endothelial cells through an increase in proinflammatory fatty acids and increased expression of proinflammatory genes and chemokines. This is the first report describing an in vitro endothelial cell model of AKT2 pathway dysregulation as in the disease familial partial lipodystrophy and in hypoinsulinemic hypoketotic hypoglycemia.

## 4. Material and Methods

### 4.1. Human PSC Cell Culture and Endothelial Cell Differentiation

Pluripotent stem cells (PSC) from HUES9 with WT AKT2, the knockout deletion (AKT2 KO), or the constitutive mutation (AKT2 E17K), engineered previously [4], were grown in feeder-free adherent culture in mTeSR1 media (StemCell Technologies, Vancouver, BC, Canada) on plates precoated with hESC-qualified Matrigel (Corning, New York, NY, USA). Differentiation into ECs and culture conditions postdifferentiation were carried out as described previously [9,17]. Briefly, PSCs were dissociated using Accutase (Innovative Cell Technologies, San Diego, CA, USA) and plated on growth-factor-reduced Matrigel (BD Biosciences) in mTeSR1 with 10 μM ROCK inhibitor Y-27632 (BioTechne, Minneapolis, MN, USA). After 24 h, the medium was replaced with priming medium consisting of N2B27 medium (1:1 mixture of DMEM:F12 (1:1) with Neurobasal media supplemented with N2, B27, and Glutamax—all Life Technologies, Carlsbad, CA) with 1 μM CP21R7 (Roche, Basel, Switzerland) and 25 ng/mL BMP4 (BioTechne, Minneapolis, MN, USA). After 3 days, the priming medium was replaced by induction medium consisting of StemPro-34 SFM medium (Life Technologies, Carlsbad, CA) supplemented with 200 ng/mL VEGF (Peprotech, Rocky Hill, NJ, USA) and 2 μM forskolin (Merck, Darmstadt, Germany). On day 6 of differentiation, the cell monolayer was dissociated with Accutase and MACS-separated using CD144 (VE-Cadherin) MicroBeads (Miltenyi Biotec, Bergisch Gladbach, Germany). CD144-positive sorted endothelial cells were replated on human-fibronectin-coated (Merck, Darmstadt, Germany) dishes at a density of 25,000 cells sqcm in EC Expansion Medium, consisting of StemPro-34 SFM (Thermo Fisher Scientific, Waltham, MA, USA) supplemented with 50 ng/mL VEGF-A (Peprotech, Rocky Hill, NJ, USA). Cells at confluence were cryopreserved.

### 4.2. Western Blotting

ECs were cultured in six-well plates until confluency, washed with 1× cold PBS^−−^, and lysed using 100 μL lysis buffer (1× Laemmli (BioRad, Hercules, CA, USA)) in RIPA buffer (Thermo Fisher Scientific, Waltham, MA, USA), 2.5% ß-mercaptoethanol (Merck, Darmstadt, Germany), and one tablet of PhosphoSTOP and ProteaseInhibitor, EDTA-free, per 10 mL (Roche, Basel, Switzerland)). Cells were immediately scraped off the plate and transferred to precooled tubes on ice. Next, samples were mixed by vortexing, heated to 95 °C for 5 min, and cooled to 4 °C. Protein concentration was measured using a Pierce 660 nm protein assay kit (Thermo Fisher Scientific, Waltham, MA, USA). Levels of AKT2 and GAPDH proteins were quantified using an automated size resolving capillary electrophoresis system (WES kit, BioTechne, Minneapolis, MN, USA). All procedures were performed according to the manufacturer’s instructions. Briefly, 8 μL of 0.2 mg/mL of homogenate was mixed with 2 μL of 5× fluorescent master mix and heated at 95 °C for 5 min. The samples, blocking reagent, primary antibody (AKT2 (D6G4) Rabbit mAb or GAPDH (14C10) Rabbit mAb (Cell Signaling Technologies, Danvers, MA, USA)), anti-rabbit secondary antibody, chemiluminescent substrate, and wash buffer were loaded into a microplate provided with a 12–230 kDa WES kit (BioTechne, Minneapolis, MN, USA). Separation, immunodetection, and signal quantification was performed automatically using the default plate settings in Compass software 4.0 (BioTechne, Minneapolis, MN, USA).

### 4.3. Measurement of Cellular Oxygen Consumption Rate

ECs were plated at 1 × 10^5^ cells per well in fibronectin-coated XF 24-well cell culture microplates (Agilent, Santa Clara, CA, USA). Prior to the assay, cells were incubated in Seahorse XF Base Medium supplemented with 1 mM pyruvate, 2 mM glutamine, and 10 mM glucose for 1 h without CO_2_. The oxygen consumption rate was measured using an XF24 extracellular flux analyzer (Agilent, Santa Clara, CA, USA) in real time and plotted as a function of time, normalized to protein concentration (pM/min/mg). Mitochondrial respiration was profiled by injecting the perturbation drugs oligomycin (1 μM) and FCCP (2 μM).

### 4.4. Metabolic Profiling

On fibronectin-coated lumox plates (ultrathin, gas-permeable film base plates, Sarstedt AG (Nuembrecht, Germany), 0.6 million cells were seeded per replicates in full EGM-2 media (Lonza). For each cell line, 12 replicates were seeded, the medium was changed after 48 h, and cells were harvested after a total of 72 h. Afterwards, 500 μL of spent medium was quickly centrifuged and the supernatant was transferred into a fresh tube and snap-frozen in liquid nitrogen. Lumox films were excised, washed quickly, transferred into tubes, and snap-frozen. MxP^®^ Broad Profiling was performed using gas chromatography (GC) MS (6890 GC coupled to an 5973 MS-System, and liquid chromatography (LC) MS/MS (1100 high-performance LC (HPLC) (all Agilent, Santa Clara, CA, USA) coupled to an API4000 MS/MS-System (Life Technologies, CA, USA) was used as previously described in detail [28]. In short, proteins were removed from supernatant samples (500 μL) and spin-filtered (Ultrafree^®^-MC 5.0 μm, Merck, Darmstadt, Vanderburgh, IN, USA) for 5 min. Filtrates were diluted with water, extracted with dichloromethane/ethanol (2:1), and phase separation was achieved by centrifugation for 5 min at 12,000 rpm. Polar and nonpolar fractions were separated. For LC-MS/MS analyses, fractions were dried under reduced pressure and reconstituted in appropriate elution solvents. HPLC was performed by gradient elution using methanol/water/formic acid on reverse-phase separation columns. Mass spectrometric detection technology was applied as described in patent US719632314, which allows multiple reaction monitoring in parallel for a full-screen analysis. For GC-MS analyses, the nonpolar fraction was treated with methanol under acidic conditions to yield the fatty acid methyl esters derived from both free fatty acids and hydrolyzed complex lipids. The polar and nonpolar fractions were further derivatized with O-methyl-hydroxyamine hydrochloride (20 mg/mL in pyridine) to convert oxo groups to O-methyloximes and subsequently with a silylating agent (N-methyl-N-(trimethylsilyl) trifluoroacetamide) before GC-MS analysis. Data were normalized to the median of reference samples which were derived from a pool of equivalent volume aliquots of all samples to account for inter- and intra-instrumental variation. Pooled samples for intracellular profiling were generated by combining additionally collected samples in equal numbers from all groups. To account for varying cell densities in the different cell cultures used for this study, all metabolite profiling data (including those from spent media samples) were further normalized to the protein concentrations from cell pellet samples. Pooled reference samples were run in parallel throughout and used to assess analytical process variability. In the metabolism study, variability within sample groups was evaluated by calculating the standard deviation of log_10_-transformed data. ANOVA was used as follows: the difference in metabolite levels between cell lines was determined by calculating the “ratio of medians” for each of the metabolites derived from the MxP^®^ platforms. In addition, *p*-values, *t*-values, and false discovery rates (FDR/*q*-values) [29] were determined. Calculations were performed separately for cell lysates and supernatant samples. Cell lines were compared against each other and supernatant data were compared to a blank medium (medium that has not been in contact with the cells). ANOVA was conducted using the statistical software R with the package nlme.

### 4.5. Multiplexed Sandwich Immunoassay

Supernatants were removed from confluent cell cultures after 24 h of growth and diluted with assay reagent to be in the linear range (2-, 20-, 50-, or 100-fold) depending on the analyte. Analytes were measured through multiplex sandwich immunoassays using a chemiluminescence reader (Cirascan, Quanterix Corp., Billerica, MA, USA). Concentrations were determined using a standard dilution curve of the respective analytes. Averages were calculated as relative values of WT cells.

### 4.6. Leukocyte Adhesion Assay

HESC-ECs (20,000 per well) were seeded in fibronectin-coated clear-bottom black plates (Corning, New York, NY, USA) and left to attach and reach confluence for 48 h at 37 °C under 5% CO_2_. Cells were treated or not with 50 ng/mL TNF-α. HL-60 cells were cultured in suspension in RPMI 1640 containing 10% FCS and 2 mM l-glutamine. For the assay, cells were washed twice with 1× PBS^−−^ and then adjusted to 1 × 10^6^ cells/mL in 1×PBS^−−^. Next, cells were labeled with 2 μM Calcein-AM (Life Technologies, Carlsbad, CA, USA) for 30 min at 37 °C in the dark, and excess Calcein-AM was washed out by two washes with 1× PBS^−−^. Next, cells were resuspended and adjusted to 1.1 × 10^6^ cells/mL with HBSS + 0.1% BSA. To each well, 50 μL of this cell suspension was added and left to attach for 1 h at 37 °C. Cells were washed gently four times with 1× PBS^++^ and fixed with ice-cold 4% paraformaldehyde for 15 min at 4 °C. Finally, cells were washed twice with 1× PBS^++^. The number of attached HL-60 cells per well was imaged using Operetta (Perkin Elmer, Waltham, MA, USA) and quantified with Columbus software 2.6 (Perkin Elmer, Waltham, MA, USA). Nine replicates were carried out per genotype.

### 4.7. Statistical Analysis

Prism 7.0 (GraphPad, San Diego, CA, USA) was used to create charts and perform statistical analyses. Unless mentioned otherwise, statistical analysis was performed using an unpaired, two-tailed Student’s *t*-test. For all column graphs, data are represented as mean ± SD, and *p*-values < 0.05 were considered significant.

## Figures and Tables

**Table 1:** Quantification of the difference in metabolite levels in supernatant and/or cell lysates between hPSC-ECs carrying AKT2 mutations (AKT2 E17K and AKT2 KO) and WT.

Analytes Analyzed in:	Supernatant						Cell Lysate		
Comparison between experimental groups	E17K	KO	E17K	KO	WT	E17K	E17K	KO	E17K
	WT	WT	KO	fresh media	fresh media	fresh media	WT	WT	KO
Significance Thresholds									
*p* < 0.1	22	23	34	39	38	35	51	39	63
*p* < 0.05	15	12	15	31	28	32	39	29	39
*p* < 0.01	4	3	7	22	20	21	21	12	15

**Table 2:** Metabolites (listed by class) that were significantly different in supernatant and/or cell lysates between hPSC-ECs carrying AKT2 mutations (AKT2 E17K and AKT2 KO) and WT.

Analytes Analyzed in:	Supernatant						Cell Lysate		
Comparison between experimental groups	E17K	KO	E17K	KO	WT	E17K	E17K	KO	E17K
	WT	WT	KO	Fresh media	Fresh media	Fresh media	WT	WT	KO
ONTOLOGY	**number of significantly changed analytes**								
Amino acids	4	1	1	6	6	6	1	2	7
Amino acids related	0	1	1	4	3	4	0	0	0
Carbohydrates and related	3	4	2	4	4	6	0	0	0
Complex lipids, fatty acids and related	3	1	0	0	0	2	13	8	11
Energy metabolism and related	1	1	1	2	3	2	0	0	2
Hormones, signal substances and related	0	0	0	1	1	1	0	0	0
Miscellaneous	0	0	0	0	0	0	1	1	2
Nucleobases and related	0	0	0	3	3	3	0	0	0
Unknown	4	4	9	10	8	7	24	18	15
Vitamins, cofactors and related	0	0	1	1	0	1	0	0	2
Total number of analytes	15	12	15	31	28	32	39	29	39

## Supplementary Materials

Supplementary materials can be found at https://www.mdpi.com/1422-0067/20/24/6201/s1.
