# Supplementary material for: Modeling the Effects of Severe Metabolic Disease by Genome Editing of hPSC-Derived Endothelial Cells Reveals an Inflammatory Phenotype
Source: Int J Mol Sci. 2019 Dec 9;20(24):6201. doi: 10.3390/ijms20246201 (PMC6940871; doi:10.3390/ijms20246201)
Supplement: Supplementary file 1 [file ijms-20-06201-s001.zip › ijms-656960-final sup/ijms-656960-Supplementary Figure and Table Legends.docx]

**Supplementary Materials and Methods**

**Cell lysis and RNA isolation.** Media was removed and cells were washed once with 1× PBS^--^, followed by lysis with RLT buffer containing 1% ß-mercapthoethanol (Merck, Darmstadt, Germany). RNA isolation from cultured cells was performed using an RNeasy mini kit (Qiagen, Hilden, Germany) with DNAse I digestion.

**Quantitative real-time PCR.** Gene expression was quantified by TaqMan RT-PCR with the Quant Studio 12k Flex real-time PCR system using the comparative C_T_ method [1] . Each reaction was multiplexed with RPLP0 (4326314E). Expression was normalized to the expression of the reference gene. TaqMan probe/primer sets for human PTGS2 (Hs00153133_m1), CCL5 (Hs00982282_m1), S100A4 (Hs00243202_m1), ADM (Hs00969450_g1), PECAM1 (Hs01065282_m1), AKT1 (Hs00178289_m1), and GLUT4 (SLC2A4, Hs00168966_m1) were designed by Life Technologies (Carlsbad, CA, USA).

**Real-time electric cell substrate impedance.** Endothelial cell proliferation was measured in real time using xCELLigence real time cell analysis (RTCA) and 96-well array plates (E-plate Cardio 96, both ACEA Biosciences, San Diego, CA, USA) at 10,000 Hz frequency. For first 30 h, cellular proliferation was observed every 15 min, and then every 1 h as reflected by the cell index (CI). Plates were coated with 100 μL of fibronectin (25 μg/mL, Corning, New York, NY, USA) for 30 min at room temperature (RT), then replaced with complete media. Afterwards, hPSC-ECs were seeded (10,000 cells per well) in complete media, left to attach for 30 h, and then starved in starvation media (EBM-2, 1% FBS, 1% l-glutamine, LifeLine, Frederick, MD, USA) either for 15 h or to the end of the assay. After 15 h of starvation, one set of cells was treated with vascular endothelial growth factor A (VEGFA; 50 ng/mL, Peprotech, Rocky Hill, NJ, USA) to the end of the assay. All data were normalized to the first impedance background measurement.

**Cell migration assay.** The cell migration assay was carried out using a monolayer TScratch wound-healing assay as described previously [2, 3]. Briefly, a cross-shaped scratch was made into a confluent hPSC-EC monolayer. After washing, full EGM-2 media was added, and images of crosses were taken both immediately and 20 h later (a minimum of 12 replicate scratches were measured). The percentage of cells in the scratched zone was determined using Wimasis scratch image analysis software (https://www.wimasis.com/).

**Cell proliferation assay.** The cell proliferation assay was carried out as described previously [4]. Briefly, ECs were seeded onto black clear-bottom fibronectin-coated 96-well plates (Costar) and left to attach for 24 h in complete EGM-2 media. Next, ECs were starved overnight in starvation media. Finally, cells were cultured for 72 h with or without VEGFA (50 ng/mL, Peprotech, Rocky Hill, NJ, USA ) and viable cells were quantified by measuring hydrolysis of the fluorogenic substrate 4-methylumbelliferyl heptanoate (MUH, Merck, Darmstadt, Germany) (18 wells per condition) [5].

**Capillary tube formation assay**. The capillary tube formation assay was conducted as described previously [6] with minor modifications. Matrigel (Corning, New York, NY, USA) was added to 96-well plates (40 µL/well) and was allowed to solidify for 30 min at 37 °C. ECs were seeded on top of the Matrigel and incubated for 12 h as sextuplicates. Images were acquired with a digital camera (Zeiss) mounted on an inverted microscope (Zeiss). The length of tube-like structures per image was measured using ImageJ 1.52.

**Supplementary Figure and Table Legends**

**Supplementary Figure 1.** qRT-PCR measurements of gene expression of metabolic and control genes. Gene expression as determined by qRT-PCR presented in AKT2 KO and AKT2 E17K for (**A**) endothelial cell markers CD31, NOTCH1, and NOS3; genes involved in insulin signaling pathway: (**B**) AKT1 and (**C**) GLUT4. Each column is the mean of triplicates ± SD. *** *p* < 0.001.

**Supplementary Figure 2.** Further evidence of metabolic dysregulation of hPSC-derived ECs carrying AKT2 mutations (AKT2 E17K) or deletion (AKT2 KO). Abundance of (**A**) glucose-6-P, glycerol, and glycerol-3-P, as well as (**B**) arginine, tryptophan, glycine, proline, lysine, and glutamate from six replicates of each hPSC-EC (WT, AKT2 E17K, and AKT2 KO) as measured by mass spectrometry in cellular lysates ± SD.

**Supplementary Figure 3.** qRT-PCR measurements of gene expression for control genes as evidence of the inflammatory phenotype of AKT2 mutant/deleted hPSC-ECs. Gene expression as determined by qRT-PCR presented as (**A**) proinflammatory genes induced in both AKT2 E17K and AKT2 KO, (**B**) an anti-inflammatory gene downregulated in AKT2 E17K, and (**C**) a proinflammatory gene upregulated in AKT2 KO. Each column is the mean of triplicates ± SD. *** *p* < 0.001, ** *p* < 0.01.

**Supplementary Figure 4.** HL-60 adhesion to endothelial cells after TNF-alpha stimulation, EC proliferation, EC migration and EC tube formation are not affected by AKT2 mutation or deletion. In each experiment, PSC-ECs (wild-type, AKT2 E17K and AKT2 KO) are compared. Adhesion of HL-60 cells to endothelial cells after 4 h stimulation with TNF-alpha (50 ng/mL). (B) Proliferation of endothelial cells was evaluated by electric cell-substrate impedance after starvation without (left panel) or with (right panel) 50 ng/mL VEGFA stimulation. Mean ±SD from 16 replicates is presented. (C) Relative viable cell numbers were measured using a MUH assay after culturing in starvation media without or with VEGFA stimulation. Columns represent 18 replicates ±SD. (D) Cell migration was evaluated using a TScratch assay in complete media. Columns represent 12 replicates ±SD. (E) Capillary tube formation was evaluated by seeding cells on matrigel and taking images of tube-like structures. Columns represent average tube-length on at least 8 images ±SD.

**Supplementary Table 1.** Complete list of the metabolites that were measured in cell lysates, including ratios of cell types, as indicated, and corresponding *p*- and *q*-values.

**Supplementary Table 2.** Complete list of the metabolites that were measured in cell supernatants, including ratios of cell types, as indicated, and corresponding *p*- and *q*-values.

Reference

1. Schmittgen, T. D.; Livak, K. J. Analyzing real-time PCR data by the comparative C(T) method. *Nat. Protoc.* **2008**, *3*, 1101–1108.

2. Roudnicky, F.; Poyet, C.; Wild, P.; Krampitz, S.; Negrini, F.; Huggenberger, R.; Rogler, A.; Stohr, R.; Hartmann, A.; Provenzano, M., et al. Endocan is upregulated on tumor vessels in invasive bladder cancer where it mediates VEGF-A-induced angiogenesis. *Cancer Res.* **2013**, *73*, 1097–1106.

3. Geback, T.; Schulz, M. M.; Koumoutsakos, P.; Detmar, M. TScratch: A novel and simple software tool for automated analysis of monolayer wound healing assays. *BioTechniques* **2009**, *46*, 265–274.

4. Roudnicky, F.; Yoon, S. Y.; Poghosyan, S.; Schwager, S.; Poyet, C.; Vella, G.; Bachmann, S. B.; Karaman, S.; Shin, J. W.; Otto, V. I., et al. Alternative transcription of a shorter, non-anti-angiogenic thrombospondin-2 variant in cancer-associated blood vessels. **2018**, *37*, 2573–2585.

5. Detmar, M.; Imcke, E.; Ruszczak, Z.; Orfanos, C. E. Effects of recombinant tumor necrosis factor-alpha on cultured microvascular endothelial cells derived from human dermis. *J. Invest. Dermatol.* **1990,** 95, 219–222.
