# Supplementary material for: Modeling the Effects of Severe Metabolic Disease by Genome Editing of hPSC-Derived Endothelial Cells Reveals an Inflammatory Phenotype
Source: Int J Mol Sci. 2019 Dec 9;20(24):6201. doi: 10.3390/ijms20246201 (PMC6940871; doi:10.3390/ijms20246201)
Supplement: Supplementary file 1 [file ijms-20-06201-s001.zip › ijms-656960-final sup/Supplementary_Table_1.pdf]

	Comparison in	cell lysate								
	CELL LINE group	E17K	KO	E17K	E17K	KO	E17K	E17K	KO	E17K
	CELL LINE reference	WT	WT	KO	WT	WT	KO	WT	WT	KO
ONTOLOGY2_NAME	METABOLITE_NAME	Ratio	Ratio	Ratio	p.value	p.value	p.value	q.value	q.value	q.value
Amino acids, acidic	Aspartate	1.14	1.01	1.13	0.2586	0.9118	0.3037	0.5427	0.9774	0.5325
Amino acids, acidic	Glutamate	1.08	1.27	0.85	0.3931	0.0214	0.0968	0.6032	0.1581	0.2655
Amino acids, aromatic	Phenylalanine	1.23	0.89	1.39	0.2807	0.5135	0.0991	0.5487	0.7865	0.2675
Amino acids, aromatic	Tryptophan	1.12	0.79	1.41	0.4929	0.1630	0.0509	0.6925	0.4742	0.2086
Amino acids, aromatic	Tyrosine	1.21	0.82	1.47	0.2979	0.2671	0.0463	0.5566	0.6219	0.2071
Amino acids, basic	Arginine	1.18	0.70	1.70	0.3687	0.0654	0.0131	0.5912	0.3316	0.1123
Amino acids, basic	Glutamine	1.28	0.95	1.36	0.3872	0.8462	0.2958	0.6032	0.9654	0.5293
Amino acids, basic	Lysine	1.30	0.69	1.88	0.2768	0.1425	0.0208	0.5487	0.4571	0.1315
Amino acids, branched chain	Isoleucine	1.15	0.77	1.50	0.4155	0.1386	0.0337	0.6090	0.4531	0.1594
Amino acids, branched chain	Leucine	1.14	0.78	1.47	0.4153	0.1327	0.0322	0.6090	0.4512	0.1566
Amino acids, branched chain	Valine	1.17	0.75	1.56	0.3349	0.0971	0.0174	0.5715	0.4180	0.1233
Amino acids, neutral	Alanine	1.03	0.99	1.04	0.8388	0.9140	0.7559	0.9156	0.9774	0.8652
Amino acids, neutral	Glycine	0.93	0.94	1.00	0.0011	0.0017	0.8035	0.0210	0.0470	0.8870
Amino acids, neutral	Proline	0.91	0.85	1.07	0.2429	0.0658	0.4292	0.5269	0.3316	0.6345
Amino acids, neutral	Serine	1.07	0.89	1.21	0.6359	0.4213	0.2141	0.7885	0.7079	0.4232
Amino acids, neutral	Threonine	1.18	0.84	1.40	0.3429	0.3172	0.0677	0.5715	0.6445	0.2249
Amino acids, S-containing	Methionine	1.10	0.79	1.40	0.5119	0.1319	0.0427	0.6976	0.4512	0.1963
Amino acids, S-containing	Taurine	1.09	0.93	1.18	0.6447	0.7004	0.4040	0.7885	0.9021	0.6295
Amino acid metabolites	5-Oxoproline (additional: Folic acid, Glutamate, Glutamine)	1.12	0.90	1.25	0.3255	0.3402	0.0692	0.5715	0.6497	0.2249
Amino acid metabolites	beta-Alanine (additional: Pantothenic acid)	0.91	0.77	1.18	0.5122	0.0908	0.2609	0.6976	0.4063	0.4895
Creatine metabolism	Creatine	0.95	0.89	1.06	0.5155	0.1616	0.4219	0.6976	0.4742	0.6345
Creatine metabolism	Creatinine	1.07	0.71	1.50	0.8625	0.3747	0.2943	0.9221	0.6901	0.5293
Creatine metabolism	Phosphocreatine	0.55	0.56	0.99	0.1943	0.2039	0.9750	0.5067	0.5333	0.9807
Urea cycle and related	Ornithine (additional: Arginine, Citrulline)	1.19	0.90	1.32	0.4401	0.6413	0.2280	0.6395	0.8722	0.4405
Monosaccharides	Glucose	1.20	0.69	1.74	0.6031	0.3086	0.1386	0.7709	0.6445	0.3318
Polyols	myo-Inositol	0.97	0.90	1.07	0.7213	0.2597	0.4270	0.8285	0.6131	0.6345
Ceramides	Ceramide (d18:1,C24:0)	0.88	0.99	0.89	0.0123	0.8245	0.0181	0.0870	0.9621	0.1233
Ceramides	Ceramide (d18:1,C24:1) (additional: Ceramide (d18:2,C24:0))	0.89	0.99	0.90	0.0041	0.8108	0.0061	0.0405	0.9621	0.0797
Cholesterol and related	Cholesterol, free	1.02	1.05	0.97	0.7011	0.3518	0.5738	0.8172	0.6645	0.7617
Cholesterol and related	Cholesterol, total	0.92	0.95	0.97	0.4011	0.5784	0.7685	0.6089	0.8550	0.8652
Cholesterylesters	Cholesterylester C16:0	1.12	0.88	1.27	0.7023	0.6748	0.4284	0.8172	0.8929	0.6345
Cholesterylesters	Cholesterylester C16:1	1.03	0.91	1.13	0.8738	0.6074	0.5039	0.9284	0.8677	0.6908
Cholesterylesters	Cholesterylester C18:1	0.95	1.20	0.80	0.6646	0.1383	0.0667	0.8071	0.4531	0.2249
Cholesterylesters	Cholesterylester C18:2	1.05	0.78	1.34	0.8047	0.2094	0.1417	0.9000	0.5390	0.3345
Cholesterylesters	Cholesterylester C20:4	1.36	0.88	1.55	0.0591	0.3913	0.0128	0.2391	0.6901	0.1123
Fatty acids, mono-unsaturated	Elaidic acid (C18:trans[9]1)	0.90	0.96	0.93	0.1373	0.5959	0.3108	0.4064	0.8677	0.5325
Fatty acids, mono-unsaturated	Oleic acid (C18:cis[9]1)	1.02	0.94	1.08	0.8527	0.4656	0.3649	0.9175	0.7328	0.5853
Fatty acids, poly-unsaturated	Arachidonic acid (C20:cis[5,8,11,14]4)	1.15	1.19	0.97	0.0434	0.0164	0.5822	0.1992	0.1339	0.7617
Fatty acids, poly-unsaturated	Eicosapentaenoic acid (C20:cis[5,8,11,14,17]5)	0.80	0.85	0.94	0.0312	0.1024	0.4954	0.1567	0.4180	0.6908

Fatty acids, poly-unsaturated	Linoleic acid (C18:cis[9,12]2)	1.06	1.00	1.06	0.6808	0.9716	0.6552	0.8150	0.9774	0.7992
Fatty acids, saturated	Lignoceric acid (C24:0)	0.90	1.03	0.88	0.1662	0.7231	0.0928	0.4631	0.9174	0.2627
Fatty acids, saturated	Myristic acid (C14:0)	0.73	1.10	0.66	0.0532	0.5379	0.0179	0.2205	0.8092	0.1233
Fatty acids, saturated	Palmitic acid (C16:0)	0.99	1.15	0.86	0.9075	0.0663	0.0543	0.9407	0.3316	0.2086
Fatty acids, saturated	Stearic acid (C18:0)	1.08	1.06	1.01	0.2069	0.2736	0.8523	0.5118	0.6286	0.9189
Fatty alcohols	Glycerol, lipid fraction	0.89	1.15	0.78	0.0317	0.0134	0.0003	0.1567	0.1339	0.0245
Glycolipids	Galactose, lipid fraction	0.94	0.99	0.96	0.2452	0.7912	0.3585	0.5269	0.9539	0.5853
Glycolipids	Glucose, lipid fraction	1.06	1.30	0.81	0.7613	0.1649	0.2630	0.8686	0.4742	0.4895
Glycolipids	myo-Inositol, lipid fraction	0.96	1.03	0.94	0.4595	0.5478	0.1943	0.6620	0.8168	0.3927
Glycolipids	N-Acetylneuraminic acid, lipid fraction	0.83	1.03	0.81	0.2745	0.8754	0.2172	0.5487	0.9774	0.4245
Lipid precursors	Glycerol-3-phosphate, polar fraction	1.07	1.36	0.79	0.5978	0.0296	0.0745	0.7699	0.1934	0.2261
Lipid precursors	O-Phosphoethanolamine	0.95	1.15	0.83	0.6990	0.3130	0.1748	0.8172	0.6445	0.3785
Lysophosphatidylcholines	Lysophosphatidylcholine (C16:0)	0.98	1.02	0.96	0.5776	0.6030	0.2919	0.7543	0.8677	0.5293
Lysophosphatidylcholines	Lysophosphatidylcholine (C18:0)	1.00	1.06	0.94	0.9955	0.1826	0.1810	0.9955	0.5006	0.3798
Lysophosphatidylcholines	Lysophosphatidylcholine (C20:4)	1.08	1.15	0.94	0.1912	0.0358	0.3306	0.5067	0.2172	0.5565
Lysophosphatidylethanolamines	Lysophosphatidylethanolamine (C18:0)	0.88	0.99	0.90	0.0323	0.7690	0.0540	0.1567	0.9473	0.2086
Lysophosphatidylethanolamines	Lysophosphatidylethanolamine (C18:1)	0.92	1.01	0.92	0.1957	0.8936	0.1585	0.5067	0.9774	0.3593
Phosphatidylcholines	Phosphatidylcholine (C16:0,C18:2)	1.04	1.00	1.03	0.0462	0.8093	0.0701	0.2013	0.9621	0.2249
Phosphatidylcholines	Phosphatidylcholine (C16:0,C20:4) (additional: Phosphatidylcholin	1.29	1.16	1.11	3.33E-05	0.0022	0.0129	0.0047	0.0545	0.1123
Phosphatidylcholines	Phosphatidylcholine (C16:0,C22:6) (additional: Phosphatidylcholin	1.02	1.09	0.94	0.7760	0.2453	0.3684	0.8794	0.5873	0.5853
Phosphatidylcholines	Phosphatidylcholine (C16:1,C18:2)	1.01	1.05	0.96	0.8402	0.1243	0.1720	0.9156	0.4401	0.3785
Phosphatidylcholines	Phosphatidylcholine (C18:0,C18:1)	1.02	0.95	1.07	0.2433	0.0394	0.0048	0.5269	0.2307	0.0720
Phosphatidylcholines	Phosphatidylcholine (C18:0,C18:2)	0.97	0.99	0.98	0.3737	0.6918	0.6123	0.5912	0.9021	0.7886
Phosphatidylcholines	Phosphatidylcholine (C18:0,C20:4)	1.17	1.02	1.14	0.0002	0.4407	0.0006	0.0076	0.7274	0.0245
Phosphatidylcholines	Phosphatidylcholine (C18:0,C22:6)	1.06	1.00	1.05	0.0792	0.9182	0.0942	0.2854	0.9774	0.2627
Phosphatidylcholines	Phosphatidylcholine No 02	1.14	1.07	1.06	5.48E-05	0.0046	0.0123	0.0047	0.0707	0.1123
Phospholipid metabolites	Glycerol phosphate, lipid fraction	0.96	1.06	0.90	0.4762	0.3694	0.1239	0.6797	0.6901	0.3095
Phospholipid metabolites	Phosphate, lipid fraction	1.08	0.99	1.09	0.3603	0.9298	0.3657	0.5833	0.9774	0.5853
Sphingolipids	erythro-Sphingosine (d18:1) (additional: Sphingolipids)	0.95	1.14	0.83	0.8375	0.6378	0.5023	0.9156	0.8722	0.6908
Sphingolipids	threo-Sphingosine (d18:1) (additional: Sphingolipids)	1.02	1.17	0.87	0.9271	0.5067	0.5651	0.9552	0.7830	0.7617
Sphingomyelins	Sphingomyelin (d18:1,C16:0)	1.08	1.00	1.08	0.0079	0.9986	0.0079	0.0649	0.9986	0.0899
Sphingomyelins	Sphingomyelin (d18:1,C18:0) (additional: Sphingomyelin (d16:1,C2	1.10	0.98	1.12	0.0017	0.4247	0.0005	0.0270	0.7079	0.0245
Sphingomyelins	Sphingomyelin (d18:1,C23:0)	0.91	0.98	0.93	0.0936	0.6557	0.1938	0.3126	0.8847	0.3927
Sphingomyelins	Sphingomyelin (d18:1,C24:0)	0.90	0.91	0.99	0.0024	0.0043	0.7308	0.0290	0.0707	0.8509
Citrate cycle	Fumarate (additional: Maleate)	1.02	0.99	1.03	0.9527	0.9619	0.9148	0.9640	0.9774	0.9425
Citrate cycle	Malate	0.86	0.92	0.93	0.3562	0.6156	0.6629	0.5822	0.8697	0.7992
Citrate cycle	Succinate	1.25	1.08	1.15	0.3422	0.7301	0.5351	0.5715	0.9194	0.7278
Glycolysis/Gluconeogenesis	Fructose-1,6-diphosphate	1.16	1.24	0.94	0.3310	0.1678	0.6515	0.5715	0.4742	0.7992
Glycolysis/Gluconeogenesis	Glucose-6-phosphate (additional: Fructose-6-phosphate, myo-Inos	0.83	1.20	0.69	0.2232	0.2453	0.0297	0.5269	0.5873	0.1531
Glycolysis/Gluconeogenesis	Lactate	0.99	0.95	1.04	0.9013	0.6682	0.7597	0.9407	0.8929	0.8652
Glycolysis/Gluconeogenesis	Pyruvate (additional: Phosphoenolpyruvate (PEP))	1.28	0.78	1.64	0.2163	0.2124	0.0242	0.5178	0.5390	0.1386
Miscellaneous	Phosphate (inorganic and from organic phosphates)	1.05	1.08	0.97	0.4074	0.2171	0.6606	0.6090	0.5429	0.7992
Miscellaneous	Pyrophosphate (PPi) (additional: Phosphate (inorganic and from oı	0.91	1.36	0.67	0.6180	0.1242	0.0531	0.7783	0.4401	0.2086

Miscellaneous	Serine, lipid fraction	0.94	0.97	0.97	0.2903	0.5352	0.6455	0.5529	0.8092	0.7992
Polyamines	Spermine	0.87	0.89	0.98	0.2346	0.2934	0.8793	0.5269	0.6445	0.9284
Nucleotides	Adenosine diphosphate (ADP) (additional: Adenosine triphosphate	0.76	1.01	0.76	0.1290	0.9471	0.1157	0.3916	0.9774	0.2979
Nucleotides	Adenosine triphosphate (ATP)	0.65	1.19	0.55	0.0806	0.4451	0.0209	0.2854	0.7276	0.1315
Purine metabolism	Adenine	1.01	0.99	1.03	0.9527	0.9404	0.8934	0.9640	0.9774	0.9312
Purine metabolism	Inosine	0.76	**0.35**	**2.19**	0.4798	0.0173	0.0607	0.6797	0.1339	0.2195
Pyrimidine metabolism	Uridine	1.45	1.04	1.40	0.0023	0.7003	0.0043	0.0290	0.9021	0.0720
Redox status	Glutathione / Glutathione disulfide	**2.22**	1.78	1.25	0.5112	0.6331	0.8540	0.6976	0.8722	0.9189
Unknown lipid	Unknown lipid (29971443)	1.05	0.98	1.07	0.0673	0.4572	0.0180	0.2543	0.7328	0.1233
Unknown lipid	Unknown lipid (29971882)	0.93	1.37	0.68	0.5171	0.0151	0.0049	0.6976	0.1339	0.0720
Unknown lipid	Unknown lipid (29971886)	1.17	1.12	1.04	0.0027	0.0148	0.3360	0.0290	0.1339	0.5600
Unknown lipid	Unknown lipid (69970017)	0.93	1.03	0.91	0.0138	0.2317	0.0017	0.0938	0.5709	0.0476
Unknown lipid	Unknown lipid (69970020)	1.07	1.18	0.91	0.0109	2.90E-05	0.0022	0.0842	0.0049	0.0529
Unknown lipid	Unknown lipid (69970121)	0.96	0.97	0.99	0.3098	0.3834	0.8775	0.5664	0.6901	0.9284
Unknown lipid	Unknown lipid (69970127)	1.07	0.99	1.08	0.0456	0.8431	0.0322	0.2013	0.9654	0.1566
Unknown lipid	Unknown lipid (69970150)	1.03	1.12	0.92	0.2811	0.0036	0.0247	0.5487	0.0681	0.1386
Unknown lipid	Unknown lipid (69970151)	0.85	0.87	0.98	0.0003	0.0008	0.4762	0.0076	0.0267	0.6803
Unknown lipid	Unknown lipid (69970153)	0.94	0.91	1.03	0.2472	0.1003	0.5739	0.5269	0.4180	0.7617
Unknown lipid	Unknown lipid (69970154)	0.99	1.00	0.99	0.8271	0.9348	0.8911	0.9156	0.9774	0.9312
Unknown lipid	Unknown lipid (69970512)	0.90	0.93	0.97	0.1497	0.3259	0.6095	0.4315	0.6445	0.7886
Unknown lipid	Unknown lipid (69970534)	0.87	1.10	0.79	0.2077	0.4044	0.0657	0.5118	0.6901	0.2249
Unknown lipid	Unknown lipid (69970538)	1.33	0.71	1.88	0.3939	0.3087	0.0780	0.6032	0.6445	0.2326
Unknown lipid	Unknown lipid (69970546)	1.02	0.99	1.03	0.6800	0.7774	0.4908	0.8150	0.9481	0.6908
Unknown lipid	Unknown lipid (69970547)	0.95	1.02	0.93	0.1818	0.6190	0.0800	0.4984	0.8697	0.2344
Unknown lipid	Unknown lipid (69970550)	0.99	0.93	1.06	0.6973	0.0610	0.1181	0.8172	0.3316	0.2998
Unknown lipid	Unknown lipid (69970551)	0.94	1.01	0.93	0.2067	0.8595	0.1566	0.5118	0.9741	0.3593
Unknown lipid	Unknown lipid (69970553)	1.11	1.08	1.03	0.0854	0.1916	0.6223	0.2962	0.5171	0.7954
Unknown lipid	Unknown lipid (69970558)	0.99	1.11	0.90	0.9349	0.2850	0.2529	0.9574	0.6445	0.4830
Unknown lipid	Unknown lipid (69970574)	0.89	0.90	1.00	0.3756	0.3966	0.9675	0.5912	0.6901	0.9790
Unknown lipid	Unknown lipid (69970597)	0.95	0.98	0.96	0.2440	0.7170	0.4073	0.5269	0.9165	0.6295
Unknown lipid	Unknown lipid (69970610)	0.92	0.96	0.97	0.1094	0.3371	0.4711	0.3444	0.6497	0.6803
Unknown lipid	Unknown lipid (69970611)	0.91	0.95	0.96	0.1078	0.3319	0.4725	0.3444	0.6486	0.6803
Unknown lipid	Unknown lipid (69970615)	0.98	0.97	1.01	0.5260	0.3194	0.7042	0.7041	0.6445	0.8313
Unknown lipid	Unknown lipid (69970619)	1.06	1.00	1.06	0.0629	0.9270	0.0736	0.2488	0.9774	0.2261
Unknown lipid	Unknown lipid (69970620)	0.98	1.06	0.93	0.6130	0.1163	0.0489	0.7776	0.4401	0.2086
Unknown lipid	Unknown lipid (69970621)	1.03	1.09	0.94	0.3478	0.0128	0.0688	0.5741	0.1339	0.2249
Unknown lipid	Unknown lipid (69970623)	1.03	1.00	1.03	0.3427	0.9488	0.3132	0.5715	0.9774	0.5325
Unknown lipid	Unknown lipid (69970625)	1.12	1.23	0.91	0.0164	0.0003	0.0278	0.0990	0.0175	0.1475
Unknown lipid	Unknown lipid (69970626)	1.16	1.13	1.02	0.0077	0.0168	0.6549	0.0649	0.1339	0.7992
Unknown lipid	Unknown lipid (69970627)	1.30	1.16	1.12	0.0001	0.0059	0.0232	0.0060	0.0829	0.1386
Unknown lipid	Unknown lipid (69970629)	1.10	1.15	0.96	0.0047	0.0003	0.1084	0.0444	0.0175	0.2837
Unknown lipid	Unknown lipid (69970631)	1.16	1.11	1.04	0.0026	0.0161	0.3040	0.0290	0.1339	0.5325
Unknown lipid	Unknown lipid (69970632)	0.98	0.99	0.99	0.6430	0.7808	0.8515	0.7885	0.9481	0.9189

Unknown lipid	Unknown lipid (69970633)	0.90	0.93	0.96	0.0023	0.0231	0.1964	0.0290	0.1638	0.3927
Unknown lipid	Unknown lipid (69970636)	1.03	1.04	0.99	0.7902	0.7386	0.9457	0.8896	0.9233	0.9685
Unknown lipid	Unknown lipid (69970638)	1.29	1.32	0.97	0.0175	0.0101	0.7534	0.0990	0.1316	0.8652
Unknown lipid	Unknown lipid (69970639)	0.95	0.94	1.01	0.1967	0.1520	0.8702	0.5067	0.4742	0.9284
Unknown lipid	Unknown lipid (69970641)	1.02	0.99	1.03	0.7066	0.9231	0.6372	0.8172	0.9774	0.7992
Unknown lipid	Unknown lipid (69970642)	1.03	0.96	1.07	0.5805	0.3967	0.1759	0.7543	0.6901	0.3785
Unknown lipid	Unknown lipid (69970658)	1.09	1.07	1.03	0.0236	0.0896	0.4493	0.1254	0.4063	0.6584
Unknown lipid	Unknown lipid (69970800)	0.91	0.96	0.95	0.1524	0.4934	0.4216	0.4319	0.7695	0.6345
Unknown lipid	Unknown lipid (69970801)	0.88	0.99	0.89	0.0659	0.8921	0.0831	0.2543	0.9774	0.2395
Unknown lipid	Unknown lipid (69970802)	1.15	1.26	0.91	0.0158	0.0006	0.0726	0.0990	0.0261	0.2261
Unknown lipid	Unknown lipid (69970803)	0.90	1.06	0.85	0.0080	0.1237	0.0006	0.0649	0.4401	0.0245
Unknown lipid	Unknown lipid (69970805)	1.13	1.13	1.00	0.1202	0.1178	0.9902	0.3716	0.4401	0.9902
Unknown lipid	Unknown lipid (69970806)	0.81	0.82	0.98	0.0201	0.0286	0.8429	0.1105	0.1934	0.9189
Unknown lipid	Unknown lipid (69970813)	1.11	1.13	0.98	0.0707	0.0337	0.6708	0.2613	0.2119	0.8030
Unknown lipid	Unknown lipid (69970814)	1.08	1.10	0.98	0.3052	0.2018	0.7811	0.5639	0.5333	0.8736
Unknown lipid	Unknown lipid (69970815)	1.06	1.08	0.98	0.2480	0.1568	0.7675	0.5269	0.4742	0.8652
Unknown lipid	Unknown lipid (69970816)	0.88	0.97	0.91	0.0148	0.4597	0.0552	0.0969	0.7328	0.2086
Unknown lipid	Unknown lipid (69970817)	0.87	0.94	0.93	0.0017	0.0633	0.0571	0.0270	0.3316	0.2111
Unknown lipid	Unknown lipid (69970818)	1.14	1.05	1.08	0.0006	0.0783	0.0132	0.0137	0.3802	0.1123
Unknown lipid	Unknown lipid (69970819)	1.08	1.01	1.06	0.1066	0.7639	0.1737	0.3444	0.9473	0.3785
Unknown lipid	Unknown lipid (69970820)	1.13	0.98	1.15	0.0169	0.6031	0.0068	0.0990	0.8677	0.0823
Unknown lipid	Unknown lipid (69970823)	0.91	1.01	0.90	0.0118	0.6293	0.0051	0.0870	0.8722	0.0720
Unknown lipid	Unknown lipid (69970824)	0.95	0.97	0.98	0.2127	0.4631	0.5825	0.5166	0.7328	0.7617
Unknown lipid	Unknown lipid (69970825)	0.95	0.92	1.02	0.3185	0.1702	0.6774	0.5715	0.4742	0.8053
Unknown lipid	Unknown lipid (69970831)	1.08	1.14	0.95	0.0408	0.0029	0.1502	0.1929	0.0624	0.3497
Unknown lipid	Unknown lipid (69970832)	0.84	0.97	0.86	0.0003	0.4060	0.0010	0.0076	0.6901	0.0327
Unknown lipid	Unknown lipid (69970841)	0.94	0.90	1.05	0.5812	0.3102	0.6286	0.7543	0.6445	0.7975
Unknown lipid	Unknown lipid (69970843)	0.81	0.88	0.92	0.0009	0.0166	0.1085	0.0198	0.1339	0.2837
Unknown polar	Unknown polar (39970411)	1.17	1.08	1.09	0.0512	0.3261	0.2649	0.2178	0.6445	0.4895
Unknown polar	Unknown polar (39971564)	1.33	0.91	1.46	0.2422	0.6775	0.1256	0.5269	0.8929	0.3095
Unknown polar	Unknown polar (39972124)	0.59	1.02	0.58	0.1386	0.9596	0.1277	0.4064	0.9774	0.3101
Unknown polar	Unknown polar (39972157)	0.88	1.21	0.73	0.2770	0.1033	0.0147	0.5487	0.4180	0.1192
Unknown polar	Unknown polar (59970813)	0.90	1.16	0.78	0.5780	0.4052	0.1793	0.7543	0.6901	0.3798
Unknown polar	Unknown polar (59970919)	0.98	1.05	0.93	0.9066	0.8192	0.7300	0.9407	0.9621	0.8509
Acyl-carriers and related	Pantothenic acid	1.28	0.78	1.66	0.0938	0.0905	0.0039	0.3126	0.4063	0.0720
Amino-carriers and related	Pyridoxine	1.25	0.82	1.52	0.2750	0.3246	0.0533	0.5487	0.6445	0.2086
Carotenoids	beta-Carotene	0.76	0.98	0.78	0.3249	0.9278	0.3682	0.5715	0.9774	0.5853
Redox-carrier and related	Coenzyme Q10	0.99	0.94	1.06	0.8985	0.1538	0.1882	0.9407	0.4742	0.3901
Redox-carrier and related	Coenzyme Q9	0.85	0.99	0.86	0.2927	0.9666	0.3107	0.5529	0.9774	0.5325
Redox-carrier and related	Flavine adenine dinucleotide (FAD)	0.91	0.98	0.93	0.2840	0.8429	0.3749	0.5487	0.9654	0.5901
Redox-carrier and related	Glutathione (GSH)	1.56	1.23	1.27	0.6365	0.8262	0.7986	0.7885	0.9621	0.8870
Redox-carrier and related	Glutathione disulfide (GSSG)	0.70	0.69	1.02	0.3355	0.3151	0.9643	0.5715	0.6445	0.9790
Redox-carrier and related	Nicotinamide	1.17	0.72	1.62	0.4154	0.1058	0.0253	0.6090	0.4184	0.1386

Redox-carrier and related	Nicotinamide adenine dinucleotide (NAD)	1.00	1.01	0.99	0.9748	0.8735	0.8983	0.9806	0.9774	0.9312
Redox-carrier and related	Nicotinamide adenine dinucleotide, reduced (NADH)	0.97	0.89	1.10	0.8485	0.3912	0.4998	0.9175	0.6901	0.6908
