# Supplementary material for: Modeling the Effects of Severe Metabolic Disease by Genome Editing of hPSC-Derived Endothelial Cells Reveals an Inflammatory Phenotype
Source: Int J Mol Sci. 2019 Dec 9;20(24):6201. doi: 10.3390/ijms20246201 (PMC6940871; doi:10.3390/ijms20246201)
Supplement: Supplementary file 1 [file ijms-20-06201-s001.zip › ijms-656960-final sup/Supplementary_Table_2.pdf]

				Comparison in	supernatant																	
				CELL LINE group	E17K	KO	E17K	KO	WT	E17K	E17K	KO	E17K	KO	WT	E17K	E17K	KO	E17K	KO	WT	E17K
				CELL LINE reference	WT	WT	KO	FM	FM	FM	WT	WT	KO	FM	FM	FM	WT	WT	KO	FM	FM	FM
MET_CHEM_NO	CAS_UID	ONTOLOGY1_NAME	ONTOLOGY2_NAME	METABOLITE_NAME	Ratio	Ratio	Ratio	Ratio	Ratio	Ratio	p.value	p.value	p.value	p.value	p.value	p.value	q.value	q.value	q.value	q.value	q.value	q.value
39980072	CAS-56-84-8	Amino acids	Amino acids, acidic	Aspartate	0.95	1.00	0.95	1.01	1.01	0.96	0.0795	0.9329	0.0681	0.7883	0.8501	0.1330	0.4270	0.9515	0.2783	0.9022	0.9634	0.3478
39980088	CAS-56-86-0	Amino acids	Amino acids, acidic	Glutamate	0.90	0.94	0.96	1.50	1.60	1.45	0.0059	0.0591	0.2540	5.68E-09	8.54E-10	1.91E-08	0.2533	0.3815	0.4626	9.65E-08	1.74E-08	2.79E-07
39980073	CAS-63-91-2	Amino acids	Amino acids, aromatic	Phenylalanine	0.99	0.98	1.01	1.00	1.02	1.00	0.0324	0.0062	0.4116	0.6215	0.0224	0.7671	0.2965	0.2094	0.6460	0.7987	0.0846	0.8598
19980035	CAS-54-12-6 , CAS-73-22-3	Amino acids	Amino acids, aromatic	Tryptophan	1.17	1.11	1.05	1.03	0.93	1.08	0.0245	0.1030	0.4512	0.6323	0.2639	0.2412	0.2965	0.4157	0.6793	0.7987	0.5079	0.4895
19980173	CAS-60-18-4	Amino acids	Amino acids, aromatic	Tyrosine	1.04	1.06	0.98	1.00	0.94	0.98	0.6060	0.4036	0.7436	0.9779	0.4100	0.7343	0.8738	0.6859	0.8908	0.9779	0.5974	0.8477
19980043	CAS-74-79-3	Amino acids	Amino acids, basic	Arginine	0.99	0.93	1.06	0.93	1.00	0.98	0.6915	0.0539	0.1114	0.0603	0.9583	0.6692	0.8817	0.3815	0.3071	0.1709	0.9937	0.8282
39980145	CAS-70-47-3 , CAS-5794-13-8	Amino acids	Amino acids, basic	Asparagine	0.96	1.10	0.87	1.03	0.94	0.90	0.4461	0.0786	0.0179	0.5461	0.2536	0.0730	0.8738	0.3815	0.1659	0.7684	0.4974	0.2258
39980466	CAS-71-00-1	Amino acids	Amino acids, basic	Histidine	1.05	0.98	1.08	0.97	0.99	1.04	0.4705	0.7227	0.2883	0.6207	0.8738	0.5954	0.8738	0.8672	0.4983	0.7987	0.9789	0.7786
39980013	CAS-56-87-1 , CAS-39665-12-8	Amino acids	Amino acids, basic	Lysine	0.99	1.01	0.98	1.02	1.01	1.01	0.3756	0.3985	0.0957	0.0360	0.1579	0.5443	0.7981	0.6859	0.2997	0.1223	0.3428	0.7326
39980074	CAS-73-32-5	Amino acids	Amino acids, branched chain	Isoleucine	0.99	1.01	0.98	0.99	0.98	0.97	0.6339	0.2059	0.0913	0.5989	0.0937	0.0402	0.8738	0.5677	0.2997	0.7987	0.2515	0.1323
39980075	CAS-61-90-5	Amino acids	Amino acids, branched chain	Leucine	1.00	1.01	0.99	0.99	0.98	0.98	0.8830	0.2911	0.2328	0.5499	0.1208	0.0945	0.9525	0.5772	0.4481	0.7684	0.3064	0.2754
39980070	CAS-72-18-4 , CAS-7004-03-7	Amino acids	Amino acids, branched chain	Valine	1.00	1.01	0.99	1.00	0.99	0.99	0.8872	0.2864	0.2308	0.8438	0.4095	0.3403	0.9525	0.5772	0.4481	0.9253	0.5974	0.6073
39980003	CAS-56-41-7	Amino acids	Amino acids, neutral	Alanine	0.99	1.01	0.97	1.17	1.15	1.14	0.3280	0.3002	0.0554	1.33E-08	4.25E-08	1.39E-07	0.7831	0.5772	0.2705	1.93E-07	5.42E-07	1.77E-06
39980004	CAS-56-40-6	Amino acids	Amino acids, neutral	Glycine	0.98	1.01	0.97	1.22	1.22	1.19	0.1906	0.6368	0.0843	2.13E-09	3.36E-09	1.32E-08	0.6031	0.8019	0.2997	4.35E-08	5.71E-08	2.25E-07
39980079	CAS-147-85-3	Amino acids	Amino acids, neutral	Proline	0.98	0.98	0.99	1.09	1.11	1.08	0.0099	0.0583	0.3739	7.78E-08	8.28E-09	2.36E-07	0.2533	0.3815	0.6053	8.81E-07	1.21E-07	2.67E-06
39980052	CAS-56-45-1	Amino acids	Amino acids, neutral	Serine	0.99	0.99	1.00	0.99	1.00	0.99	0.3547	0.2564	0.8245	0.1485	0.6946	0.2101	0.7864	0.5753	0.9246	0.3224	0.8335	0.4659
39980006	CAS-72-19-5	Amino acids	Amino acids, neutral	Threonine	1.00	1.00	1.00	1.02	1.02	1.02	0.7897	0.8155	0.9733	0.1024	0.0685	0.1083	0.9477	0.9151	0.9829	0.2547	0.2116	0.2985
39980010	CAS-52-90-4	Amino acids	Amino acids, S-containing	Cysteine (additional: Cystine)	1.00	0.99	1.01	0.92	0.93	0.93	0.9668	0.8018	0.8340	0.0104	0.0168	0.0155	0.9879	0.9151	0.9246	0.0460	0.0683	0.0658
39980008	CAS-63-68-3	Amino acids	Amino acids, S-containing	Methionine	1.00	1.01	0.99	1.00	0.99	0.99	0.6885	0.1929	0.0973	0.8001	0.3132	0.1729	0.8817	0.5623	0.2997	0.9022	0.5566	0.4101
39980009	CAS-98-79-3	Amino acids related	Amino acid metabolites	5-Oxoproline (additional: Folic acid, Glutamate, Glutamine)	0.99	0.96	1.04	0.96	1.01	1.00	0.7846	0.0753	0.1227	0.1466	0.7724	0.9771	0.9477	0.3815	0.3269	0.3224	0.9056	0.9771
39981135	CAS-107-95-9 , CAS-28854-76-4	Amino acids related	Amino acid metabolites	beta-Alanine (additional: Pantothenic acid)	1.04	1.01	1.02	1.11	1.10	1.14	0.3163	0.7045	0.5248	0.0092	0.0190	0.0027	0.7831	0.8554	0.7137	0.0428	0.0746	0.0153
39981833	CAS-816-66-0	Amino acids related	Amino acid metabolites	Ketoleucine	1.03	0.88	1.17	2.53	2.87	2.95	0.5951	0.0268	0.0092	8.96E-11	1.57E-11	1.10E-11	0.8738	0.3041	0.1359	4.57E-09	1.60E-09	1.12E-09
39980437	CAS-51-35-4	Amino acids related	Collagen metabolism	trans-4-Hydroxyproline	1.00	0.99	1.01	1.10	1.11	1.11	0.9797	0.8501	0.8701	0.0081	0.0056	0.0059	0.9894	0.9324	0.9442	0.0393	0.0287	0.0301
19980325	CAS-57-00-1	Amino acids related	Creatine metabolism	Creatine	1.04	1.04	1.00	1.11	1.07	1.12	0.3459	0.3957	0.9223	0.0345	0.1544	0.0288	0.7840	0.6859	0.9693	0.1215	0.3423	0.1077
19980321	CAS-60-27-5	Amino acids related	Creatine metabolism	Creatinine	1.26	1.16	1.09	0.97	0.83	1.05	0.0853	0.2505	0.5245	0.8235	0.1948	0.7021	0.4350	0.5753	0.7137	0.9131	0.4139	0.8425
19980327	CAS-492-27-3	Amino acids related	Tryptophan metabolism	Kynurenic acid	1.04	1.13	0.92	1.15	1.01	1.06	0.6629	0.2158	0.4090	0.2024	0.9208	0.6072	0.8750	0.5703	0.6460	0.4162	0.9886	0.7839
19980159	CAS-372-75-8	Amino acids related	Urea cycle and related	Citrulline	0.93	0.92	1.02	0.98	1.07	0.99	0.3301	0.2355	0.8209	0.7730	0.3907	0.9416	0.7831	0.5753	0.9246	0.9022	0.5948	0.9771
39980046	CAS-70-26-8	Amino acids related	Urea cycle and related	Ornithine (additional: Arginine, Citrulline)	1.05	0.99	1.06	0.99	1.00	1.05	0.2120	0.8827	0.1668	0.8528	0.9642	0.2496	0.6169	0.9350	0.3782	0.9253	0.9937	0.4895
29980428	CAS-57-13-6	Amino acids related	Urea cycle and related	Urea	1.08	1.06	1.01	0.87	0.82	0.88	0.4915	0.5404	0.9153	0.2040	0.0748	0.2604	0.8738	0.7253	0.9693	0.4162	0.2180	0.5012
39981841	CAS-3416-24-8	Carbohydrates and related	Aminosugars	Glucosamine	0.97	1.09	0.90	1.08	0.99	0.96	0.6104	0.1518	0.0610	0.2135	0.8891	0.5332	0.8738	0.5032	0.2705	0.4228	0.9789	0.7326
39981840	CAS-2636-92-2	Carbohydrates and related	Aminosugars	Mannosamine	0.97	1.01	0.96	1.05	1.04	1.01	0.5716	0.8892	0.4826	0.4324	0.5119	0.9057	0.8738	0.9350	0.6861	0.7001	0.7001	0.9623
39980021	CAS-69-79-4	Carbohydrates and related	Disaccharides	Maltose	0.83	0.72	1.15	1.61	2.22	1.85	0.1316	0.0141	0.2499	0.0016	1.27E-05	0.0002	0.5224	0.2242	0.4626	0.0095	9.99E-05	0.0011
39982122	CAS-4084-27-9	Carbohydrates and related	Monosaccharides	3-Deoxyglucosone	1.01	0.93	1.09	0.90	0.96	0.97	0.8111	0.2642	0.1813	0.0983	0.5187	0.6739	0.9509	0.5753	0.3934	0.2547	0.7001	0.8282
39980037	CAS-50-99-7 , CAS-492-62-6	Carbohydrates and related	Monosaccharides	Glucose (additional: Galactose, Gluconic acid, Mannitol)	1.03	1.01	1.02	0.87	0.86	0.88	0.1068	0.5004	0.3198	1.91E-06	7.36E-07	8.71E-06	0.4738	0.7164	0.5437	1.77E-05	6.25E-06	6.84E-05
39980019	CAS-50-69-1 , CAS-24259-59-4	Carbohydrates and related	Nucleobase related saccharides	Ribose	0.87	0.87	0.99	0.67	0.76	0.66	0.0378	0.0448	0.9295	1.88E-05	0.0008	1.63E-05	0.2965	0.3815	0.9693	0.0002	0.0052	0.0001
39980054	CAS-149-32-6	Carbohydrates and related	Polyols	Erythrol	0.98	1.04	0.94	1.01	0.97	0.95	0.1965	0.0154	0.0011	0.4592	0.0857	0.0074	0.6031	0.2242	0.0801	0.7206	0.2429	0.0357
39980014	CAS-87-89-8	Carbohydrates and related	Polyols	myo-Inositol	1.01	1.02	1.00	1.00	0.99	1.00	0.1434	0.0759	0.7194	0.7663	0.1501	0.9639	0.5224	0.3815	0.8841	0.9022	0.3422	0.9771
39980033	CAS-50-70-4	Carbohydrates and related	Polyols	Sorbitol	0.96	0.89	1.07	0.97	1.08	1.03	0.6327	0.2474	0.4843	0.7344	0.4344	0.7397	0.8738	0.5753	0.6861	0.9022	0.6240	0.8477
39980545	CAS-87-99-0	Carbohydrates and related	Polyols	Xylitol	1.07	1.24	0.86	1.19	0.96	1.03	0.4750	0.0425	0.1565	0.1133	0.6757	0.7917	0.8738	0.3815	0.3714	0.2687	0.8205	0.8777
39980822	CAS-13752-84-6	Carbohydrates and related	Sugar acids	Erythronic acid	1.06	0.97	1.10	1.03	1.07	1.13	0.2454	0.5079	0.0793	0.5685	0.2383	0.0316	0.6764	0.7164	0.2997	0.7837	0.4766	0.1077
39980111	CAS-526-95-4	Carbohydrates and related	Sugar acids	Gluconic acid (additional: Gluconolacton)	0.98	0.98	1.00	1.01	1.03	1.01	0.1560	0.2587	0.7522	0.3498	0.0564	0.5181	0.5305	0.5753	0.8908	0.6048	0.1917	0.7326
19980295	CAS-528-16-5 , CAS-6556-12-3	Carbohydrates and related	Sugar acids	Glucuronic acid	1.33	0.98	1.36	0.76	0.78	1.04	0.0342	0.8750	0.0252	0.0559	0.0739	0.7647	0.2965	0.9350	0.1791	0.1705	0.2180	0.8598
39980127	CAS-17812-24-7	Carbohydrates and related	Sugar acids	Ribonic acid	1.05	1.01	1.04	1.21	1.19	1.25	0.0091	0.3867	0.0514	3.73E-08	1.04E-07	4.16E-09	0.2533	0.6859	0.2705	4.76E-07	1.17E-06	8.48E-08
19980416	CAS-516-35-8	Complex lipids, fatty acids and related	Bile acids	Taurochenodeoxycholic acid (additional: Taurodeoxycholic acid)	0.97	1.05	0.92	1.14	1.08	1.05	0.8069	0.6739	0.5083	0.3340	0.5630	0.7272	0.9509	0.8282	0.7102	0.5976	0.7362	0.8477
19980396	CAS-81-24-3	Complex lipids, fatty acids and related	Bile acids	Taurocholic acid	1.13	1.07	1.05	1.05	0.98	1.11	0.3427	0.5890	0.6744	0.7032	0.8925	0.4385	0.7840	0.7702	0.8598	0.8747	0.9789	0.7099
19980401	CAS-57-88-5	Complex lipids, fatty acids and related	Cholesterol and related	Cholesterol, free	1.08	1.03	1.05	1.05	1.02	1.10	0.0456	0.4462	0.1804	0.2336	0.6233	0.0218	0.3302	0.7164	0.3934	0.4495	0.7754	0.0888
19980402		Complex lipids, fatty acids and related	Cholesterylesters	Cholesterylester C16:0	0.93	1.02	0.91	1.02	1.00	0.93	0.2177	0.7406	0.1257	0.7631	0.9886	0.2335	0.6169	0.8683	0.3269	0.9022	0.9937	0.4895
19980403		Complex lipids, fatty acids and related	Cholesterylesters	Cholesterylester C16:1	0.95	1.01	0.94	1.01	1.00	0.95	0.2005	0.8595	0.1498	0.8987	0.9671	0.2079	0.6031	0.9326	0.3639	0.9549	0.9937	0.4659
19980404		Complex lipids, fatty acids and related	Cholesterylesters	Cholesterylester C18:1	0.95	0.97	0.97	0.98	1.01	0.96	0.1301	0.4102	0.4603	0.5928	0.8029	0.2268	0.5224	0.6859	0.6805	0.7987	0.9277	0.4895
19980405		Complex lipids, fatty acids and related	Cholesterylesters	Cholesterylester C18:2	0.92	0.99	0.93	1.02	1.04	0.96	0.0360	0.7364	0.0682	0.5297	0.3493	0.2428	0.2965	0.8683	0.2783	0.7626	0.5841	0.4895
19980406		Complex lipids, fatty acids and related	Cholesterylesters	Cholesterylester C20:4	0.92	0.97	0.94	0.96	0.98	0.90	0.0258	0.4556	0.1062	0.2155	0.5807	0.0107	0.2965	0.7164	0.3071	0.4228	0.7497	0.0495
29980003	CAS-57-10-3	Complex lipids, fatty acids and related	Fatty acids, saturated	Palmitic acid (C16:0)	1.05	1.11	0.95	1.04	0.93	0.98	0.4920	0.1537	0.4799	0.6138	0.3756	0.8448	0.8738	0.5032	0.6861	0.7987	0.5948	0.9266
29980049		Complex lipids, fatty acids and related	Glycolipids	Galactose, lipid fraction	1.29	2.99	0.43	1.58	0.53	0.69	0.5356	0.0141	0.0595	0.2779	0.1407	0.3882	0.8738	0.2242	0.2705	0.5153	0.3338	0.6387
39980012	CAS-77-92-9	Energy metabolism and related	Citrate cycle	Citrate (additional: Isocitrate)	0.90	0.92	0.98	1.11	1.21	1.09	0.0378	0.0759	0.7117	0.0538	0.0015	0.1025	0.2965	0.3815	0.8841	0.1705	0.0087	0.2903
39980086	CAS-110-15-6	Energy metabolism and related	Citrate cycle	Succinate	1.01	1.03	0.98	0.99	0.96	0.97	0.7622	0.5077	0.7159	0.7970	0.3788	0.5481	0.9464	0.7164	0.8841	0.9022	0.5948	0.7326
39980034	CAS-57-48-7 , CAS-7660-25-5	Energy metabolism and related	Glycolysis/Gluconeogenesis	Fructose	1.00	1.01	0.99	1.02	1.01	1.01	0.9377	0.2978	0.2651	0.1009	0.4779	0.5240	0.9879	0.5772	0.4744	0.2547	0.6678	0.7326
39980076	CAS-473-81-4	Energy metabolism and related	Glycolysis/Gluconeogenesis	Glycerate	0.98	0.94	1.04	0.99	1.05	1.03	0.5886	0.1613	0.3705	0.8049	0.2731	0.5455	0.8738	0.5032	0.6053	0.9022	0.5159	0.7326
39980085	CAS-50-21-5 , CAS-113-21-3	Energy metabolism and related	Glycolysis/Gluconeogenesis	Lactate	0.88	1.02	0.87	3.50	3.45	3.05	0.0791	0.8167	0.0514	3.61E-11	4.26E-11	1.74E-10	0.4270	0.9151	0.2705	3.68E-09	1.65E-09	5.92E-09
39980002	CAS-127-17-3	Energy metabolism and related	Glycolysis/Gluconeogenesis	Pyruvate (additional: Phosphoenolpyruvate (PEP))	1.01	1.05	0.96	0.92	0.88	0.89	0.6349	0.0047	0.0122	3.53E-05	2.75E-07	5.13E-07	0.8738	0.2094	0.1542	0.0003	2.80E-06	5.18E-06
39980393	CAS-300-85-6 , CAS-625-71-8	Energy metabolism and related	Ketone bodies	3-Hydroxybutyrate	0.93	0.97	0.96	1.13	1.17	1.09	0.3193	0.6339	0.5939	0.1246	0.0546	0.2843	0.7831	0.8019	0.7867	0.2825	0.1917	0.5370
19980305	CAS-30315-93-6	Hormones, signal substances and related	Other hormones, signal substances and related	N,N-Dimethylarginine (ADMA)	1.14	0.87	1.31	1.68	1.93	2.20	0.4848	0.4702	0.1664	0.0197	0.0049	0.0013	0.8738	0.7164	0.3782	0.0836	0.0261	0.0075
19980289	CAS-50-23-7	Hormones, signal substances and related	Steroids and related	Cortisol	1.00	1.00	1.00	0.97	0.97	0.97	0.9685	0.9216	0.9530	0.3832	0.3362	0.3545	0.9879	0.9495	0.9720	0.6407	0.5716	0.6129
39980051	CAS-14265-44-2	Miscellaneous	Miscellaneous	Phosphate (inorganic and from organic phosphates)	1.02	1.08	0.94	1.03	0.96	0.97	0.6452	0.0643	0.1463	0.4193	0.2970	0.5360	0.8738	0.3815	0.3639	0.6899	0.5497	0.7326
39980619	CAS-68-94-0	Nucleobases and related	Purine metabolism	Hypoxanthine (additional: Inosine)	0.92	0.99	0.92	2.11	2.13	1.95	0.0734	0.8254	0.1092	3.68E-10	3.06E-10	1.59E-09	0.4270	0.9151	0.3071	9.37E-09	7.81E-09	4.07E-08
39981762	CAS-58-63-9	Nucleobases and related	Purine metabolism	Inosine	0.96	1.14	0.85	0.26	0.23	0.22	0.6509	0.1294	0.0570	1.62E-10	4.86E-11	3.50E-11	0.8738	0.4551	0.2705	5.49E-09	1.65E-09	1.79E-09
19980301	CAS-69-93-2	Nucleobases and related	Purine metabolism	Uric acid	1.01	1.00	1.02	0.89	0.89	0.90	0.8858	0.9435	0.8303	0.1122	0.1262	0.1592	0.9525	0.9529	0.9246	0.2687	0.3064	0.3867
19980407	CAS-58-96-8	Nucleobases and related	Pyrimidine metabolism	Uridine	0.97	0.93	1.04	1.93	2.07	2.00	0.6511	0.3592	0.6346	1.07E-06	3.08E-07	5.59E-07	0.8738	0.6661	0.8298	1.09E-05	2.86E-06	5.18E-06
39980147		Unknown	Unknown polar	Unknown polar (39980147)	0.93	1.07	0.87	1.00	0.94	0.88	0.1413	0.1628	0.0090	0.9362	0.2102	0.0145	0.5224	0.5032	0.1359	0.9646	0.4289	0.0642
39980274		Unknown	Unknown polar	Unknown polar (39980274)	1.00	0.98	1.02	1.04	1.06	1.07	0.8791	0.5175	0.4266	0.1787	0.0597	0.0454	0.9525	0.7164	0.6592	0.3796	0.1964	0.1446
39980471		Unknown	Unknown polar	Unknown polar (39980471)	0.99	0.88	1.12	0.97	1.10	1.09	0.8818	0.1903	0.2408	0.7884	0.3165	0.3862	0.9525	0.5623	0.4548	0.9022	0.5566	0.6387
39980486		Unknown	Unknown polar	Unknown polar (39980486)	1.12	0.97	1.15	0.90	0.93	1.03	0.0178	0.4628	0.0040	0.0238	0.0901	0.4816	0.2965	0.7164	0.1017	0.0969	0.2484	0.7326
39981147		Unknown	Unknown polar	Unknown polar (39981147)	0.95	0.93	1.02	0.93	1.00	0.95	0.6100	0.4944	0.8598	0.5095	0.9937	0.6211	0.8738	0.7164	0.9430	0.7626	0.9937	0.7873
39981150		Unknown	Unknown polar	Unknown polar (39981150)	1.07	0.94	1.14	0.85	0.90	0.97	0.0575	0.1016	0.0019	0.0004	0.0106	0.3453	0.3667	0.4157	0.0801	0.0025	0.0489	0.6073
39981269		Unknown	Unknown polar	Unknown polar (39981269)	1.03	0.89	1.15	1.05	1.18	1.21	0.6024	0.0494	0.0178	0.3704	0.0100	0.0036	0.8738	0.3815	0.1659	0.6297	0.0486	0.0196
39981272		Unknown	Unknown polar	Unknown polar (39981272)	0.98	0.97	1.01	1.10	1.14	1.12	0.6497	0.4843	0.8029	0.0482	0.0129	0.0305	0.8738	0.7164	0.9246	0.1587	0.0547	0.1077
39981275		Unknown	Unknown polar	Unknown polar (39981275)	1.10	0.95	1.15	0.80	0.85	0.93	0.0300	0.2180	0.0024	9.05E-05	0.0009	0.0782	0.2965	0.5703	0.0801	0.0006	0.0054	0.2345
39981564		Unknown	Unknown polar	Unknown polar (39981564)	0.94	0.85	1.11	0.93	1.10	1.03	0.3003	0.0132	0.0999	0.2613	0.1510	0.6252	0.7831	0.2242	0.2997	0.4935	0.3422	0.7873
39982017		Unknown	Unknown polar	Unknown polar (39982017)	1.00	0.93	1.07	0.89	0.96	0.96	0.9964	0.2017	0.2002	0.0585	0.4469	0.4494	0.9964	0.5677	0.4138	0.1705	0.6331	0.7163
39982043		Unknown	Unknown polar	Unknown polar (39982043)	1.04	0.98	1.07	0.88	0.90	0.94	0.4047	0.6155	0.1914	0.0248	0.0625	0.2484	0.8424	0.7947	0.4067	0.0973	0.1993	0.4895
39982045		Unknown	Unknown polar	Unknown polar (39982045)	0.96	1.09	0.88	1.00	0.91	0.88	0.4483	0.1084	0.0257	0.9287	0.1063	0.0270	0.8738	0.4157	0.1791	0.9646	0.2780	0.1058
39982076		Unknown	Unknown polar	Unknown polar (39982076)	0.88	1.04	0.85	0.79	0.76	0.67	0.0486	0.5197	0.0136	0.0018	0.0005	1.36E-05	0.3302	0.7164	0.1542	0.0101	0.0036	9.93E-05
39982091		Unknown	Unknown polar	Unknown polar (39982091)	0.98	1.10	0.89	1.07	0.97	0.95	0.7329	0.0995	0.0531	0.2971	0.5589	0.3678	0.9229	0.4157	0.2705	0.5411	0.7362	0.6252
39982095		Unknown	Unknown polar	Unknown polar (39982095)	0.92	1.07	0.86	0.66	0.62	0.57	0.2601	0.3680	0.0539	8.05E-05	1.75E-05	2.96E-06	0.6981	0.6703	0.2705	0.0006	0.0001	2.51E-05
39982104		Unknown	Unknown polar	Unknown polar (39982104)	0.93	0.88	1.06	1.05	1.20	1.12	0.3661	0.1100	0.4528	0.5272	0.0400	0.1893	0.7946	0.4157	0.6793	0.7626	0.1456	0.4389
39982117		Unknown	Unknown polar	Unknown polar (39982117)	0.96	1.10	0.87	1.00	0.92	0.88	0.6139	0.2895	0.1282	0.9589	0.3356	0.1586	0.8738	0.5772	0.3269	0.9684	0.5716	0.3867
39982196		Unknown	Unknown polar	Unknown polar (39982196)	0.97	0.86	1.12	0.87	1.00	0.97	0.4458	0.0020	0.0093	0.0035	0.9320	0.5225	0.8738	0.2003	0.1359	0.0178	0.9903	0.7326
39982221		Unknown	Unknown polar	Unknown polar (39982221)	1.00	0.90	1.11	0.82	0.91	0.91	0.9651	0.0680	0.0737	0.0035	0.1252	0.1165	0.9879	0.3815	0.2890	0.0178	0.3064	0.3128
39982224		Unknown	Unknown polar	Unknown polar (39982224)	1.13	1.05	1.08	1.00	0.96	1.08	0.1344	0.5496	0.3458	0.9551	0.6100	0.3443	0.5224	0.7281	0.5782	0.9684	0.7681	0.6073
59980874		Unknown	Unknown polar	Unknown polar (59980874)	0.91	0.90	1.00	0.98	1.08	0.98	0.5398	0.5345	0.9935	0.8924	0.6460	0.8986	0.8738	0.7253	0.9935	0.9549	0.7939	0.9623
59980876		Unknown	Unknown polar	Unknown polar (59980876)	0.80	0.64	1.25	0.75	1.17	0.94	0.2010	0.0180	0.2028	0.1220	0.3797	0.7153	0.6031	0.2298	0.4138	0.2825	0.5948	0.8477

59980878		Unknown	Unknown polar	Unknown polar (59980878)	0.93	1.06	0.88	1.13	1.06	0.99	0.7701	0.8023	0.5889	0.6343	0.8095	0.9724	0.9464	0.9151	0.7867	0.7987	0.9277	0.9771
59980879		Unknown	Unknown polar	Unknown polar (59980879)	0.98	1.12	0.88	1.23	1.10	1.08	0.8464	0.3056	0.2280	0.0773	0.3872	0.4928	0.9525	0.5772	0.4481	0.2128	0.5948	0.7326
59980881		Unknown	Unknown polar	Unknown polar (59980881)	1.58	1.07	1.48	0.68	0.64	1.01	0.0082	0.6584	0.0199	0.0286	0.0124	0.9611	0.2533	0.8189	0.1690	0.1044	0.0547	0.9771
59980885		Unknown	Unknown polar	Unknown polar (59980885)	0.99	1.17	0.85	1.16	1.00	0.99	0.9580	0.3056	0.2828	0.3446	0.9745	0.9347	0.9879	0.5772	0.4973	0.6048	0.9937	0.9771
59980889		Unknown	Unknown polar	Unknown polar (59980889)	1.04	0.70	1.49	0.65	0.93	0.97	0.8516	0.1233	0.0885	0.0793	0.7432	0.8805	0.9525	0.4491	0.2997	0.2128	0.8815	0.9555
59980901		Unknown	Unknown polar	Unknown polar (59980901)	0.73	0.72	1.02	0.88	1.22	0.89	0.0999	0.0856	0.9313	0.5006	0.3018	0.5531	0.4632	0.3968	0.9693	0.7626	0.5497	0.7326
39981436	CAS-79-83-4 , CAS-137-08-6	Vitamins, cofactors and related	Acyl-carriers and related	Pantothenic acid	1.06	0.98	1.09	0.97	1.00	1.06	0.0989	0.4864	0.0263	0.4452	0.9178	0.1370	0.4632	0.7164	0.1791	0.7096	0.9886	0.3495
19980414	CAS-82-82-6	Vitamins, cofactors and related	Amino-carriers and related	4-Pyridoxic acid	1.04	1.00	1.04	1.06	1.06	1.10	0.6691	0.9880	0.6583	0.5308	0.5216	0.3012	0.8750	0.9880	0.8500	0.7626	0.7001	0.5586
19980227	CAS-65-23-6	Vitamins, cofactors and related	Amino-carriers and related	Pyridoxine	0.98	1.04	0.94	1.10	1.05	1.03	0.5527	0.2593	0.0961	0.0286	0.2067	0.4673	0.8738	0.5753	0.2997	0.1044	0.4289	0.7326
39980083	CAS-3909-12-4	Vitamins, cofactors and related	Ascorbic acid and related	Threonic acid	1.04	1.03	1.01	1.06	1.03	1.06	0.1557	0.2651	0.7391	0.0582	0.3554	0.0317	0.5305	0.5753	0.8908	0.1705	0.5847	0.1077
19980328	CAS-98-92-0	Vitamins, cofactors and related	Redox-carrier and related	Nicotinamide	0.99	1.00	1.00	0.97	0.98	0.97	0.8708	0.9202	0.9501	0.5289	0.5920	0.4915	0.9525	0.9495	0.9720	0.7626	0.7548	0.7326
29981199	CAS-59-67-6	Vitamins, cofactors and related	Redox-carrier and related	Nicotinic acid	0.98	0.96	1.02	1.00	1.04	1.02	0.6505	0.4524	0.7598	0.9293	0.4030	0.6938	0.8738	0.7164	0.8908	0.9646	0.5974	0.8425

Statistical quantities	Explanation
Ratio	ANOVA estimate of effect on ratio scale (transformed Estimate: 10^Est.log)
p.value	p-value of ANOVA t-statistics
t.value	ANOVA t-statistics
Est.log	ANOVA contrast estimate (on log10-scale)
Std.Err	Standard error of ANOVA contrast estimate (on log10-scale)
DF	Degrees of freedom for contrast estimate
q.value	Benjamini-Hochberg multiple comparisons q-value (= False-discovery rate/FDR)

Coloring of ratios:
ratio > 1 & p-value < 0.01
ratio > 1 & 0.01 ≤ p-value < 0.05
ratio > 1 & 0.05 ≤ p-value < 0.10
ratio < 1 & 0.05 ≤ p-value < 0.10
ratio < 1 & 0.01 ≤ p-value < 0.05
ratio < 1 & p-value < 0.01

Font style of ratios:
**bold: (ratio < 0.5) or (ratio > 2**
normal: (ratio > 0.5) or (ratio < 2)

Coloring of p-values:
0.05 ≤ p < 0.10
0.01 ≤ p < 0.05
p < 0.01

METABOLITE_NAME contains an "Additional": Quantitation can be disturbed by metabolites exhibiting identical analytical characteristics with respect to quantitation method. Literature data and/or comparison with alternative methods (e.g. LC-MS/MS, GC-MS) suggest that disturbing metabolite is present at minor levels only.

CAS_UID: Numerical identifier for chemical elements, compounds and polymers assigned by the American Chemical Society (Chemical Abstracts Service).

MET_CHEM_NO: Internal metabolite identification number used by metanomics GmbH.

Data normalized to the Metabolite Median
