# Supplementary figures and images for: Modeling the Effects of Severe Metabolic Disease by Genome Editing of hPSC-Derived Endothelial Cells Reveals an Inflammatory Phenotype
Source: Int J Mol Sci. 2019 Dec 9;20(24):6201. doi: 10.3390/ijms20246201 (PMC6940871; doi:10.3390/ijms20246201)

Supplementary Figure 1. Roudnicky et al.

**A**

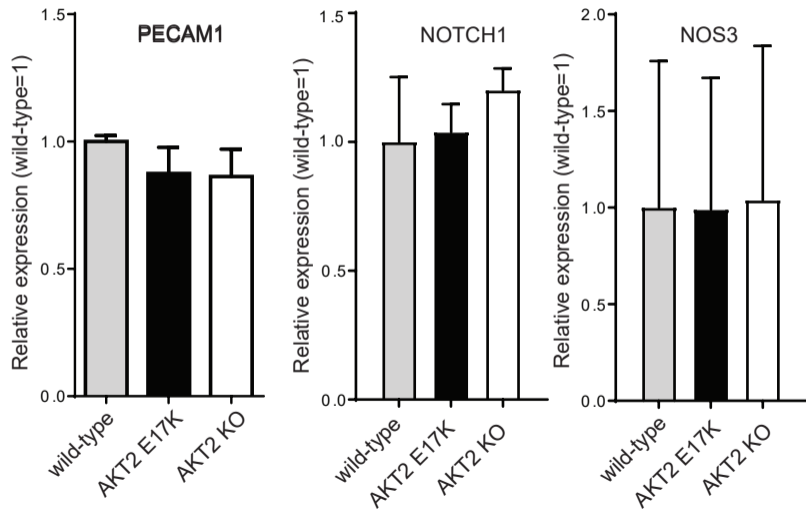

**B**

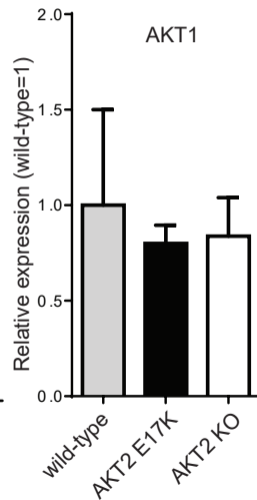

**C**

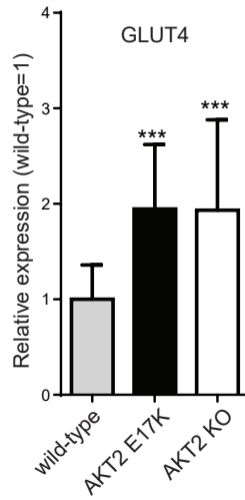

Supplement: Supplementary file 1 [file ijms-20-06201-s001.zip › ijms-656960-final sup/Supplementary_Figure1_NOTCH.pdf]

Supplementary Figure 2. Roudnicky et al.

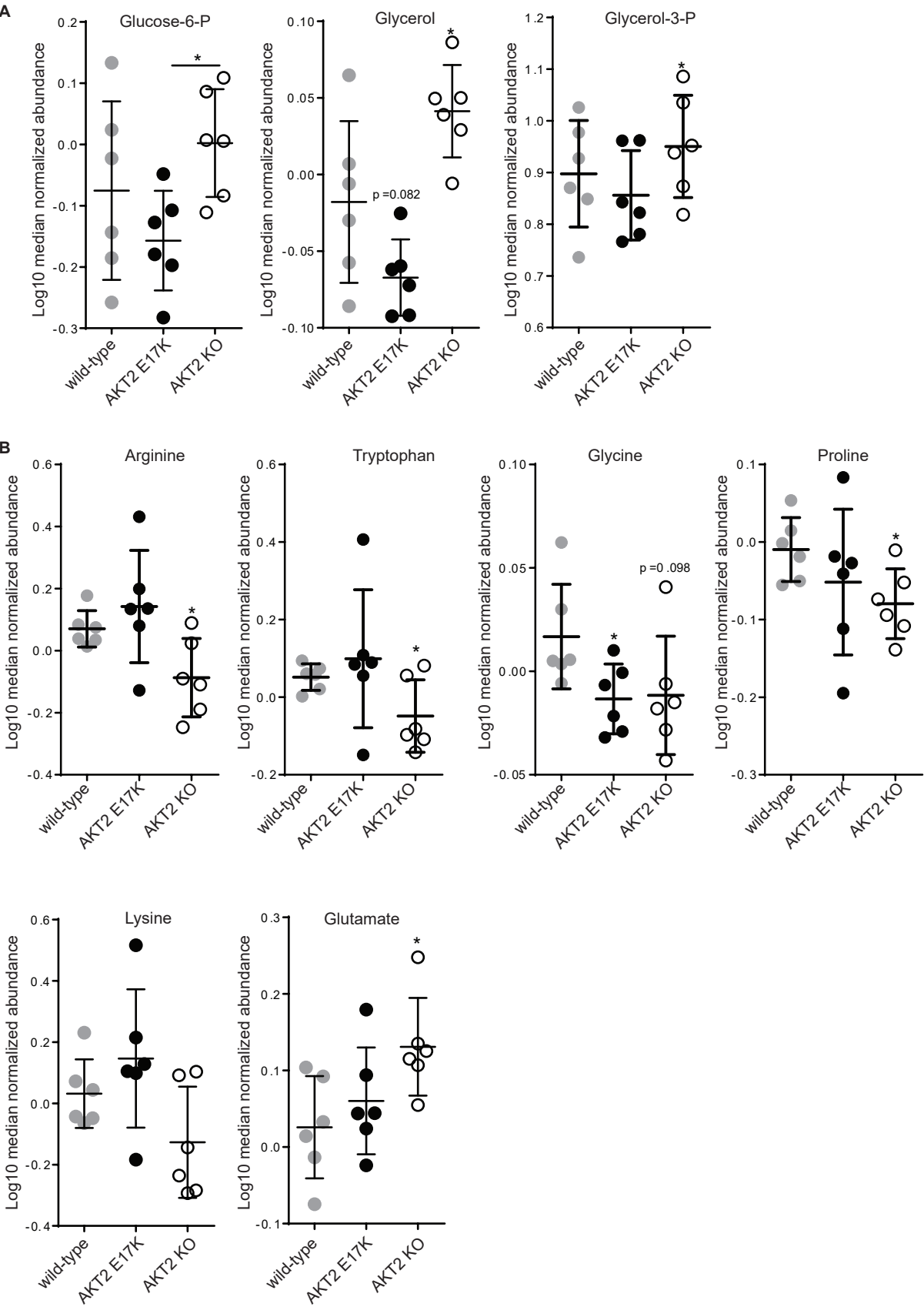

Supplement: Supplementary file 1 [file ijms-20-06201-s001.zip › ijms-656960-final sup/Supplementary_Figure2_STRes.pdf]

Supplementary Figure 3. Roudnický et al.

**A**

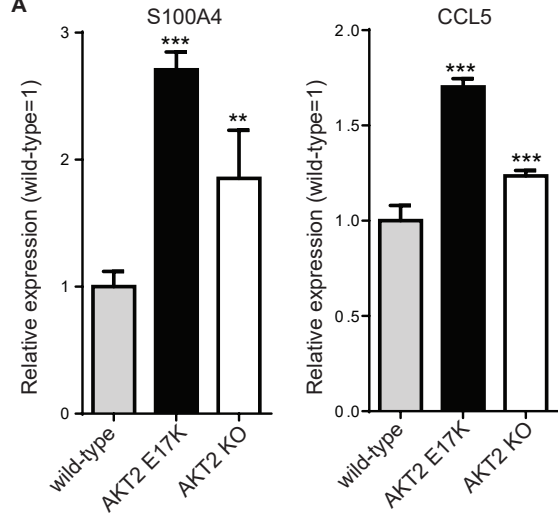

**B**

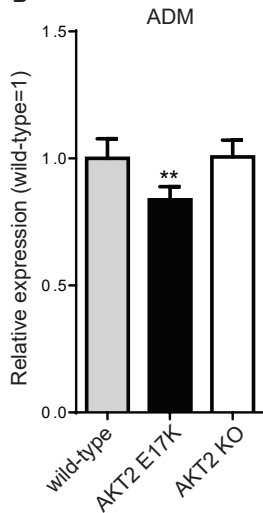

**C**

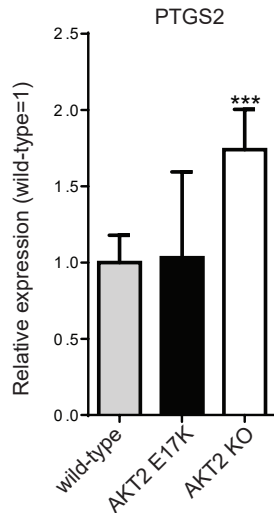

Supplement: Supplementary file 1 [file ijms-20-06201-s001.zip › ijms-656960-final sup/Supplementary_Figure3_STRes.pdf]

Supplementary Figure 4. Roudnicky et al.

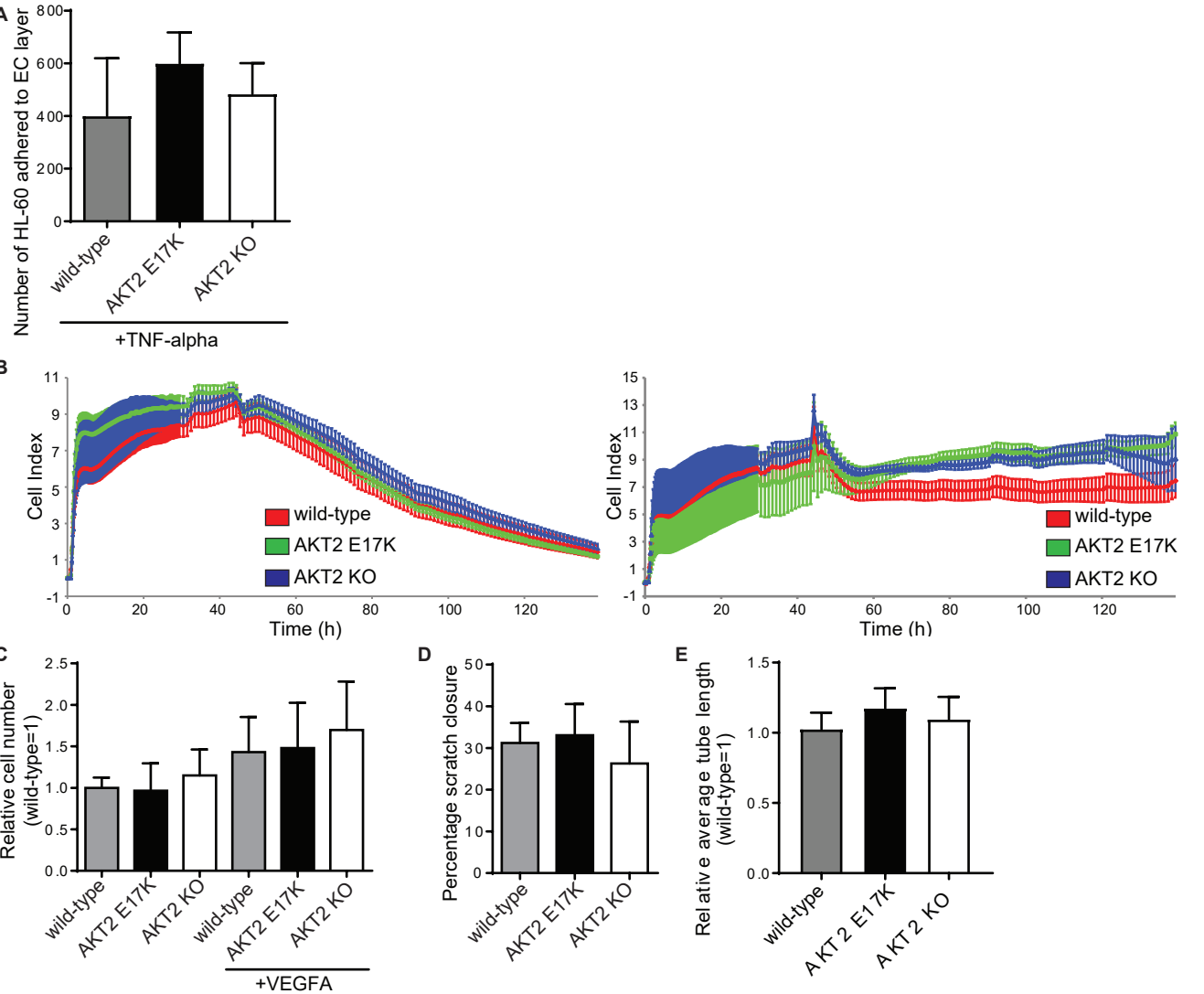

Supplement: Supplementary file 1 [file ijms-20-06201-s001.zip › ijms-656960-final sup/Supplementary_Figure4.pdf]
